# Targeted photodynamic therapy: enhancing efficacy through specific organelle engagement

**DOI:** 10.3389/fphar.2025.1667812

**Published:** 2025-08-25

**Authors:** Jiawen Tao, Zhifei Yuan, Mengjiao Zhou

**Affiliations:** ^1^ The People’s Hospital of Danyang, Affiliated Danyang Hospital of Nantong University, Danyang, China; ^2^ School of Pharmacy, Nantong University, Nantong, China

**Keywords:** photodynamic therapy, photosensitizers, reactive oxygen species, cellular organelles, subcellular organelles targeting

## Abstract

Photodynamic therapy (PDT) induces cancer cell death by utilizing photosensitizers to generate reactive oxygen species (ROS) upon light irradiation, which in turn trigger oxidative stress. However, the therapeutic efficacy of PDT is constrained by the short lifetimes and limited diffusion range of ROS, resulting in suboptimal outcomes and off-target effects. Specific organelle targeting, facilitated by rationally engineered photosensitizers and nanoplatforms with precise drug delivery capabilities that activate organelle-mediated cell death pathways, can maximize localized oxidative damage, enhance therapeutic efficacy, and minimize systemic toxicity. This review synthesizes advancements in organelle-targeted PDT, focusing on critical subcellular compartments (*e.g.*, mitochondria, lysosomes, nuclei, cell membranes, ribosome, endoplasmic reticulum, golgi apparatus, autophagosome). It systematically summarizes the structural characteristics, design strategies, targeting mechanisms, and therapeutic effects of these organelle-targeted systems, with particular emphasis on organelle-mediated cell death signaling pathways. Ultimately, current challenges, prospective opportunities, and future research directions in organelle targeting are delineated, providing a strategic framework to advance organelle-targeted PDT toward precision therapy.

## 1 Introduction

**FIGURE 1:**
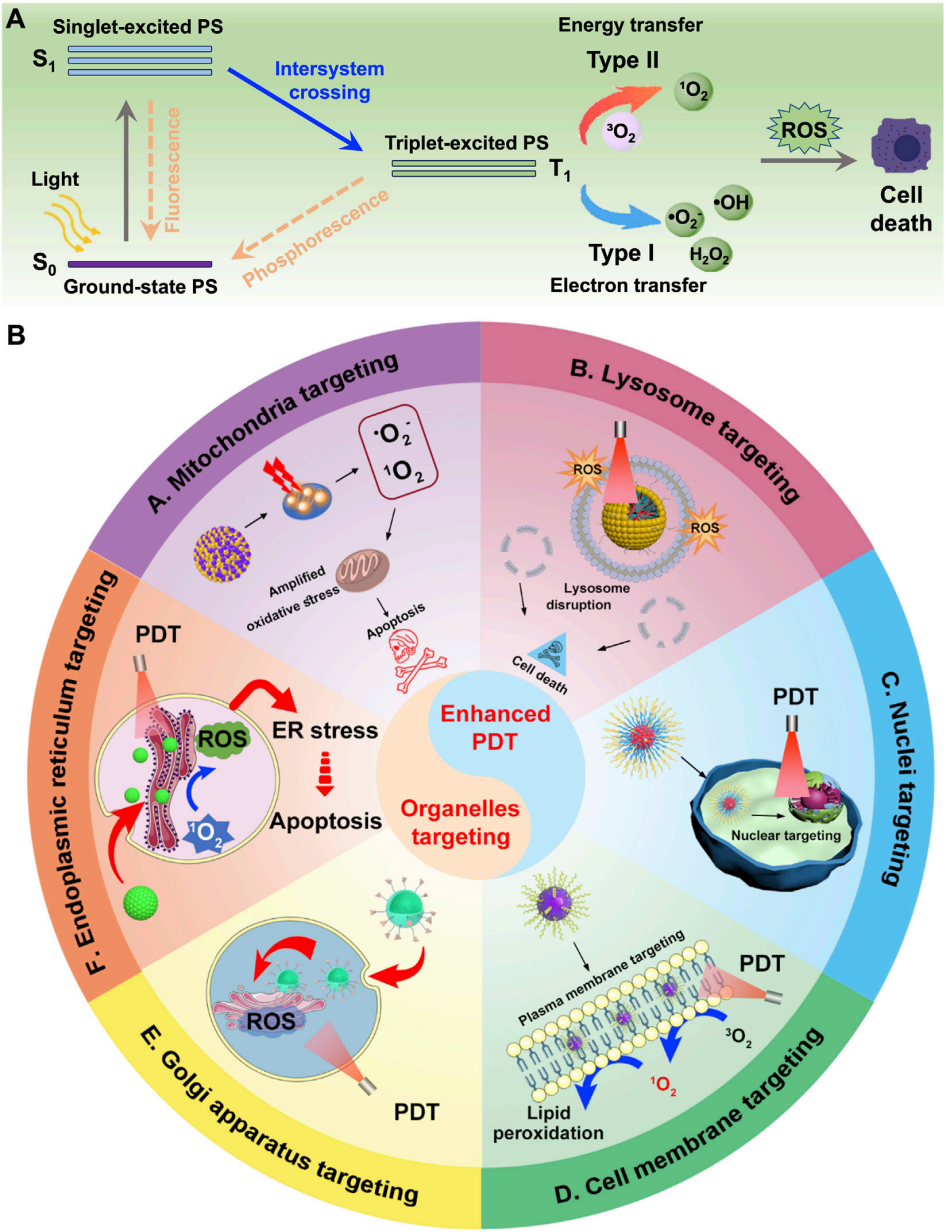
**(A)** Schematic illustration of type I and type II mechanism in the process of PDT. **(B)** Schematic illustration of different nanomedicines targeting various organelles, including mitochondria, lysosomes, nucleus, cell membrane, ER, and Golgi apparatus, to achieve enhanced PDT.

The complexity and heterogeneity of malignant tumors are major contributors to their high mortality rate, making cancer treatment a critical challenge in nanomedicine and life sciences (Bian et al., 2024; Cantallops Vilà et al., 2024). Various approaches have been developed, including surgery, chemotherapy, radiotherapy, photodynamic therapy (PDT), immunotherapy, and gene therapy (Cortés-Guiral et al., 2021; Pan Y. et al., 2022; Xie R. et al., 2023; Qian et al., 2024). Among these, PDT has gained significant attention due to its spatiotemporal precision, low cost, ease of application, and minimal toxic side effects (Li et al., 2020; Di Bartolomeo et al., 2022). Notably, unlike conventional therapies, PDT rarely causes drug resistance or severe adverse effects (Sun B. et al., 2023; Hua et al., 2024). PDT relies on three essential components: a photosensitizer, light, and oxygen (O_2_) (Kolarikova et al., 2023; Wang et al., 2023). Upon absorption of light at specific wavelengths (typically matching its absorption spectrum), the photosensitizer undergoes a transition from the ground state to an excited singlet state (S_1_), followed by intersystem crossing to form a longer-lived triplet state (T_1_) (Fay and Limmer, 2024; Kim et al., 2024). This T_1_ species then mediates the two primary mechanisms of PDT (Figure 1A). Type II PDT is defined by energy transfer from the triplet-state photosensitizer to ground-state molecular oxygen (^3^O_2_), which is converted into highly cytotoxic singlet oxygen (^1^O_2_) (Shigemitsu et al., 2022; Yu L. et al., 2024). This process is strictly O_2_-dependent, as ^1^O_2_ generation directly relies on the availability of O_2_, and the short half-life of ^1^O_2_ limits its diffusion to a narrow region around the photosensitizer. In contrast, Type I PDT is characterized by relatively low O_2_ dependence, enabling it to retain efficacy even in hypoxic tumor regions. It operates through a distinct pathway where the excited photosensitizer, typically in its T_1_, undergoes electron transfer or hydrogen atom abstraction reactions with biological substrates, such as amino acids, lipids, or nucleic acids, in the tumor microenvironment. This substrate-mediated interaction leads to the generation of a suite of reactive oxygen species (ROS), including superoxide anion radicals (•O_2_^−^), hydrogen peroxide (H_2_O_2_), and hydroxyl radicals (•OH) (Wen et al., 2022; Zhen et al., 2025). Despite these differences in ROS generation mechanisms and oxygen requirements, both Type I and Type II PDT culminate in elevated oxidative stress in the tumor microenvironment, ultimately inducing cancer cell death (Yu Y. et al., 2023; Zhang et al., 2023d).

However, we must confront an undeniable fact: despite the increasing number of studies on PDT-based anti-tumor treatments in recent years, several critical issues still need to be addressed (Sun et al., 2022). One of the most prominent challenges is the extremely short half-life (0.03–0.18 µs) and limited action range (0.01–0.02 µm) of ^1^O_2_ generated by photosensitizers (Moan and Berg, 1991; Niedre et al., 2002). As a result, the oxidative effects of PDT-induced ROS are largely confined to areas in close proximity to the photosensitizer (Austin et al., 2025). To overcome this limitation, investigating the mechanism of PDT-induced immunogenic cell death (ICD) has emerged as a potentially effective strategy (Zhang et al., 2022b; Zhang T. et al., 2023). Nevertheless, an even more promising approach may lie in further enhancing the targeting capability of photosensitizer-based nanoplatforms, particularly their ability to selectively target various subcellular organelles (Sun et al., 2024). Achieving precise subcellular localization enables targeted disruption at critical sites, triggering a systemic effect that can ultimately lead to different modes of cancer cell death, including apoptosis, necrosis, or pyroptosis (Tong et al., 2024; Wang B. et al., 2024).

Cells are the fundamental units of life, and their normal metabolism is essential for maintaining health (Finley, 2023; Sun et al., 2025). Disruptions in cellular metabolism can lead to various diseases (Plikus et al., 2021; Lee et al., 2024). Such metabolic processes rely heavily on the coordinated function and communication among intracellular organelles (König and McBride, 2024; Zimmermann et al., 2024). Common organelles include mitochondria, lysosomes, endoplasmic reticulum (ER), Golgi apparatus, endosomes, and centrosomes, each playing a distinct role in cellular function (Bornens, 2021; Celik et al., 2023; Ebner et al., 2023; Meyer and Kravic, 2024; Sun S. et al., 2024). The cell membrane, as a phospholipid bilayer structure, plays a critical role in maintaining the separation and homeostasis between the intracellular and extracellular environments (Zhu X. et al., 2024). It is particularly susceptible to oxidative attack by ROS, leading to the generation of abundant lipid peroxidation (LPO) products (Pope and Dixon, 2023; Zhu et al., 2023). This not only exacerbates membrane damage but also provides a mechanistic basis for the synergistic enhancement of PDT with ferroptosis (Pan W. et al., 2022; Chen et al., 2024). Mitochondria generate adenosine triphosphate (ATP) through aerobic respiration, providing energy for cellular activities (Dong et al., 2023; Granath-Panelo and Kajimura, 2024). Upon exposure to ROS, mitochondria experience disruption of membrane potential and functional impairment, leading to reduced O_2_ consumption (Cho and Kim, 2024). This creates a favorable environment for O_2_-dependent PDT to generate increased levels of ROS, thereby enhancing therapeutic efficacy (Cen et al., 2023). Lysosomes degrade unwanted cellular materials *via* hydrolytic enzymes (Settembre and Perera, 2024; Sweet et al., 2025). Following ROS-induced damage, the permeability of the lysosomal membrane is altered, leading to the release of intracellular cathepsins that induce cell death. This mechanism opens up new avenues for cancer therapy (Yu Q. et al., 2023; Chen F. et al., 2025). The ER is crucial for the synthesis and transport of proteins and lipids, while the Golgi apparatus modifies, sorts, and transports proteins for secretion or delivery to other organelles (Weigel et al., 2021; Tang and Ginsburg, 2023). ROS-induced oxidative stress in the ER and the reduction of immunosuppressive factor secretion by the Golgi apparatus caused by ROS both provide new opportunities and mechanistic insights for enhanced PDT (Liu M. et al., 2022; Zhang X. et al., 2022). Given these critical roles, maintaining the structural and functional integrity of organelles is vital for cell survival (Yao et al., 2021; Harapas et al., 2022). Conversely, disrupting organelle function-such as by damaging membranes, altering membrane potential, or releasing destructive enzymes-can impair cellular metabolism and trigger programmed cell death, making organelles promising targets for therapeutic interventions (Šlachtová et al., 2023; Soukar et al., 2025).

Several reviews have summarized strategies for targeting specific organelles through material design to treat diseases, primarily focusing on the development of targeting agents (Yang et al., 2022; Shao et al., 2023; Ying et al., 2023; Hong et al., 2024). In contrast, this review highlights the structural features required for designing photosensitizers that target different organelles (Figure 1B). We also evaluate which organelles are most effective targets in PDT, particularly in overcoming challenges such as tumor hypoxia, drug resistance, metastasis, and recurrence. By analyzing current studies on organelle-targeted PDT, we aim to deepen the understanding of PDT’s mechanisms in inhibiting tumor growth and provide insights for its future clinical application.

## 2 Subcellular organelle-specific targeting mechanisms for enhanced PDT

Animal cells contain a variety of structurally and functionally distinct organelles that work in concert to maintain cellular homeostasis. These organelles not only carry out essential biological processes but also serve as potential targets for therapeutic interventions, including PDT (Liu X. et al., 2022; Xu Y. et al., 2025). Targeting specific organelles with photosensitizers can enhance PDT efficacy by inducing localized damage and activating cell death pathways (Chai et al., 2022). Below is an overview of key organelles and their functional roles relevant to PDT (Table 1).

**TABLE 1:** Organelle-specific targeting strategies for enhanced PDT.

Organelle	Targeting mechanism	Biological effects	Signaling pathways	Unique advantages	References
Cell Membrane	Generate ROS, directly act on cell membrane	Disrupt ion balance; increase permeability; promote release; trigger apoptosis, necroptosis, ICD	RIPK1/RIPK3/MLKL necroptosis pathway	Bypass short ROS lifetime and reducing agents; induce LPO	Deng et al., 2022; Niu et al., 2022; Tang et al., 2023
Lysosomes	Generate ROS, alter membrane permeability	Induce cathepsin-mediated damage; activate pyroptosis; impair homeostasis	Cathepsin B damage/NLRP3 inflammasome pathway	Alternative to caspase apoptosis; enhance pyroptosis-related immunity	Luo et al., 2024; Liu et al., 2024c; Sun et al., 2024
Mitochondria	Generate ROS	Disrupt membrane potential; induce dysfunction; impair defense mechanisms	Bcl-2/Bax mitochondrial apoptosis pathway	Reduce O_2_ consumption; enhance O_2_-dependent PDT; programmed cell death induction	Khaliulin et al., 2025; Yang et al., 2023a; Yan et al., 2024
Nuclei	Generate ROS	Induce DNA fragmentation, cell cycle arrest; activate immunity	STING-mediated innate immune pathway	Directly damage genetic material; trigger antitumor immunity	Kalukula et al., 2022; Wu et al., 2024b; Feng et al., 2025
Endoplasmic reticulum	Generate ROS, induce ER stress	Upregulate CRT; enhance dendritic cell activity; trigger ICD	ER stress-mediated ICD pathway	Boost antitumor efficacy via ICD activation	Li et al., 2019; Deng et al., 2020a; Liu et al., 2022b
Golgi Apparatus	Generate ROS, impair function	Disrupt structure; release pro-inflammatory cytokines	NLRP3 inflammasome pathway	Block immuno-suppressive factors; strengthen innate/adaptive immunity	Chen et al., 2023c; Hu et al., 2023; Shi et al., 2024
Ribosome	Generate ROS, inhibit protein synthesis	Suppress translation; impair proliferation and survival	mTOR/p70S6K translational pathway	Interfere with protein production critical for growth/metastasis	Cheng et al., 2024; Elhamamsy et al., 2022; Jiao et al., 2023
Autophago-some	Generate ROS, disrupt autophagy	Impair autophagic flux; damage components; induce autophagic death	Autophagy-related pathways	Shift autophagy toward tumor death; avoid autophagy-mediated resistance	Liu et al., 2024b; Wan et al., 2019

The cell membrane acts as a selective barrier, regulating nutrient uptake and waste removal. It also plays a central role in signal transduction and intercellular communication (Bijonowski et al., 2025). Damage to the cell membrane *via* PDT can disrupt ion balance and trigger apoptosis (Fang R. H. et al., 2023). Moreover, it is well known that the therapeutic efficacy of PDT is not only limited by the short lifetime of ROS, but is also affected by intracellular reducing agents such as glutathione (GSH). The ROS generated during PDT can directly act on the cell membrane, inducing LPO and thereby achieving efficient tumor suppression (Deng et al., 2022). ROS can directly act on the cell membrane, effectively increasing membrane permeability, disrupting membrane integrity, and leading to the rapid release of intracellular contents, thereby inducing necroptosis cancer theranostics (Niu et al., 2022) and ICD (Tang et al., 2023).

**FIGURE 2:**
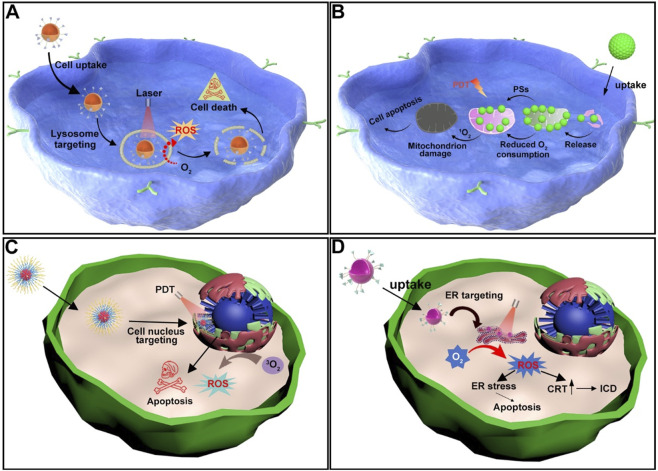
**(A)** Schematic illustration of the mechanism by which lysosome-targeting photosensitizers generate ROS upon light irradiation, leading to cancer cell death. **(B)** Schematic illustration of the mechanism by which mitochondrion-targeting photosensitizers produce ROS upon light irradiation, reducing the O_2_ consumption capacity of cancer cells and thereby enhancing PDT efficacy. **(C)** Schematic illustration of nucleus-targeting photosensitizers generating ROS upon light irradiation, resulting in apoptosis of cancer cells. **(D)** Schematic illustration of the mechanisms by which ER-targeting photosensitizers produce ROS upon light irradiation, leading to either apoptosis or ICD in cancer cells.

Lysosomes are crucial for maintaining normal cellular turnover, and have been implicated in various diseases, including cancer (Zhu et al., 2020). The integrity of the lysosomal membrane is essential for its physiological functions (Cao et al., 2021). However, studies have shown that the permeability of the lysosomal membrane can be influenced by ROS, thereby inducing a cathepsin-mediated lysosomal cell death pathway, which operates distinctly from apoptosis mediated by caspases (Figure 2A) (Wu et al., 2022; Liu X. et al., 2023). Consequently, employing photosensitizers to generate ROS with the aim of altering the integrity and functionality of lysosomal membranes may emerge as a promising therapeutic strategy for cancer treatment (Chen Z. et al., 2025). In recent years, growing evidence has confirmed that lysosomes play a critical role in the activation of pyroptosis (Zheng Z. et al., 2021; Luo et al., 2024). Specifically, when lysosomes are damaged, they release cathepsin B, which subsequently activates the NLRP3 inflammasome and caspase-1, ultimately leading to pyroptotic cell death mediated by GSDMD-N (Liu et al., 2024c; Sun et al., 2024). Moreover, recent studies have indicated that alterations in lysosomal membrane permeability can impair the ability of lysosomes to maintain cellular homeostasis, ultimately leading to lysosome-mediated cell death mechanisms (Li et al., 2023; Xie Z. et al., 2023).

The Mitochondria could generate ATP through oxidative phosphorylation and play a central role in apoptosis regulation (Kilbride and Prehn, 2013; Yadav et al., 2015; Kuznetsov et al., 2022; Khaliulin et al., 2025). ROS generated during PDT can effectively disrupt the mitochondrial membrane potential, leading to mitochondrial dysfunction (Yang F. et al., 2023). This helps prevent further O_2_ consumption by mitochondria, thereby enhancing the efficiency of O_2_-dependent PDT in producing more ROS (Figure 2B). Moreover, studies have shown that mitochondria are highly enriched with GSH, an important antioxidant molecule (Liu Y. et al., 2023). Therefore, directly targeting mitochondria may offer a more direct way to impair cellular defense mechanisms. Mitochondrial damage is one of the most effective PDT targets due to its direct involvement in programmed cell death (Yan et al., 2024).

The Nucleus can store genetic material (DNA) and controls gene expression and cell proliferation (Sabari et al., 2020; Kalukula et al., 2022). Nuclear damage induced by PDT can lead to DNA fragmentation and irreversible cell cycle arrest (Zhang et al., 2023c). Moreover, studies have shown that photoactive materials targeting the nucleus can generate ROS upon light irradiation, which directly damage nuclear DNA (Figure 2C). The resulting cytosolic DNA fragments can then work in synergy with STING agonists to activate the innate anti-tumor immune response (Wu Y. et al., 2024; Feng et al., 2025).

Photosensitive agents targeting the ER can induce significant ER stress upon light exposure, leading to the surface expression of calreticulin (CRT) (Figure 2D) (Li et al., 2019). This process stimulates the antigen-presenting capability of dendritic cells and triggers ICD, thereby suppressing cancer cell proliferation. A number of studies have demonstrated that both ER stress and ROS production play critical roles in activating intracellular signaling pathways that regulate ICD (Deng H. et al., 2020). PDT with ER-targeting specificity can further enhance the antitumor therapeutic effect.

Research indicates that the Golgi apparatus serves as a key site for the production of various immunosuppressive cytokines. The generation of these cytokines relies on the structural integrity of the Golgi, making it an effective strategy to suppress their expression by disrupting this organelle. Therefore, PDT-induced ROS can impair Golgi function and thereby prevent the release of immunosuppressive factors, which would otherwise hinder the immune response triggered by PDT (Chen Y. J. et al., 2023). This approach enhances the overall efficacy of PDT in inhibiting cancer cell proliferation. Moreover, Golgi-targeted photosensitizers can upregulate the expression of NLRP3 upon light activation, promoting the release of pro-inflammatory cytokines such as IL-1β (Hu et al., 2023). This not only strengthens innate immunity but also boosts adaptive immune responses against tumor cells. Hence, a precise phototherapy strategy targeting the Golgi apparatus offers a promising new direction for effective tumor suppression (Shi et al., 2024).

By selectively delivering photosensitizers to specific subcellular organelles, PDT can be optimized to induce site-specific damage, thereby enhancing therapeutic efficacy and addressing key challenges such as hypoxia, drug resistance, immune suppression, and tumor recurrence (Wang et al., 2021; Tian et al., 2024). A deeper understanding of the unique biological functions and characteristics of each organelle lays the groundwork for developing more effective and precisely targeted PDT strategies.

## 3 Mitochondrial targeting for enhanced PDT

Cancer treatment remains one of the key research areas in the field of biomedicine (Shen et al., 2024; Wu D. et al., 2024; Ye et al., 2024; Yu et al., 2024b). The continuous development of effective and safe nanodrug delivery systems is not only crucial for advancing cancer therapy but also an urgent and ongoing research priority. Indeed, mitochondria play a pivotal role in cancer cellular energetics (Ikeda et al., 2021). Often referred to as the “powerhouses” of eukaryotic cells, these organelles are responsible for producing the majority of ATP, the cell’s universal energy currency (Wang H. et al., 2024). This process occurs through oxidative phosphorylation, a series of reactions that take place in the inner mitochondrial membrane (Vercellino and Sazanov, 2022; Decker and Funai, 2024). Developing mitochondria-targeted nanomedicine systems can enhance the therapeutic effects of PDT by leveraging the cascade of mechanisms triggered by mitochondrial dysfunction (Ding et al., 2025). A comprehensive overview of diverse mitochondria-targeted PDT strategies, spanning GSH depletion, O_2_ generation, mitochondria respiration inhibition, and O_2_-independent radical production, are systematically summarized in Table 2.

**TABLE 2:** Comprehensive analysis of mitochondria-targeted PDT strategies.

Category	Core strategy	Effectors	Advantages	Clinical barriers	References
GSH depletion	Deplete mGSH to amplify ROS-induced damage	mGSH, GPX4, GCL/GS (GSH synthesis enzymes)	Directly enhance ROS sensitivity by removing mitochondrial antioxidant defenses, synergizes with both Type I/II PDT	Compensatory cytosolic GSH elevation, redox imbalance in normal cells	Ke et al., 2022; Huang et al., 2023
O_2_ generation	In situ O_2_ production to boost 1O_2_ yield	H_2_O_2_, ATP synthase, cardiolipin	Alleviates hypoxia locally to restore Type II PDT efficacy	Catalyst deactivation in acidic tumor, limited O_2_ diffusion	Yang et al., 2019; Li et al., 2023
Mitochondria respiration inhibition	Reduce O_2_ consumption via chain inhibition	Hexokinase-II, complex III (respiratory chain), membrane potential	Conserve endogenous O_2_ for PDT by reducing respiratory demand, direct tumor cell energy depletion	Autophagy activation via excess inhibition, normal tissue cross-reactivity	Zuo et al., 2021; Zhao et al., 2024
O_2_-independent	Generate radicals without O_2_ dependence	NQO1, lipids (LPO substrates), NADH/FADH2	Bypass O_2_ limitation in hypoxia, radicals cause irreversible mitochondrial damage	Short radical lifespan, limited two-photon depth	Fang et al., 2023a; Kuang et al., 2022

### 3.1 Targeted GSH depletion in mitochondria for enhanced PDT efficacy

Mitochondria-often referred to as the cell’s energy powerhouse-are particularly vulnerable to oxidative stress (Zhu et al., 2022). GSH is a tripeptide antioxidant present in all cells that maintains cellular redox homeostasis, with mitochondrial GSH (mGSH) playing a unique role in protecting mitochondrial DNA and respiratory chain proteins from oxidative damage (Li B. et al., 2025). Notably, mGSH is spatially and functionally distinct from cytosolic GSH: it is synthesized in the cytosol and transported into mitochondria *via* the glutamate-cystine antiporter (GC1), forming a relatively independent pool that accounts for ∼10–15% of total cellular GSH but is critical for mitochondrial redox defense (Lu et al., 2022; Xu T. et al., 2025). Tumor cells exhibit higher mGSH levels than normal cells, enabling them to scavenge ROS generated during PDT and resist oxidative stress (Song et al., 2024). Thus, selective depletion of mGSH rather than global GSH reduction avoids disrupting cytosolic redox balance and enhances PDT specificity (Zhang et al., 2025).

**FIGURE 3:**
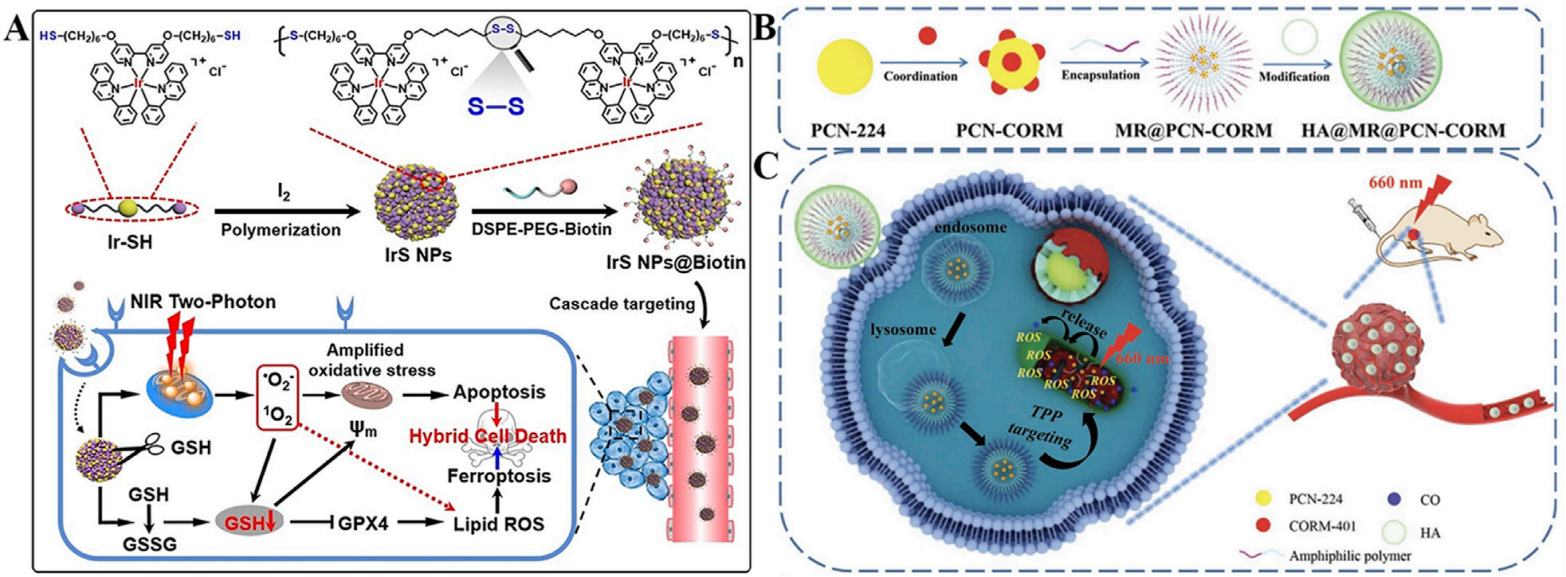
**(A)** Preparation of the IrS NPs@Biotin nanoplatform with cascade targeting and mGSH-responsive capabilities, along with its antitumor mechanism for enhanced PDT/ferroptosis. Reproduced with permission from Ref. Ke et al. (2022). Copyright ^©^ 2022 WILEY-VCH Verlag GmbH and Co. KGaA. **(B)** Preparation process of the HA@MR@PCN-CORM nanoplatform featuring hierarchical targeting and ROS-triggered CO release ability. **(C)** Schematic illustration of the multi-layered targeting HA@MR@PCN-CORM system for augmented ROS production in cancer cells. Reproduced with permission from Ref. Yang F. et al. (2023). Copyright ^©^ 2023 WILEY-VCH Verlag GmbH and Co. KGaA.

Ke *et al.* developed an iridium (III) complex containing thiols at both ends (Ir-SH) to construct self-assembled nanoparticles (IrS NPs) for targeted GSH depletion (Figure 3A) (Ke et al., 2022). Surface modification with Biotin, a small molecule ligand that specifically binds to overexpressed biotin receptors on tumor cells, enabling active targeting cellular uptake. Intrinsic lipophilic cationic properties of Ir-SH, which facilitates accumulation in mitochondria *via* electrostatic attraction to the negatively charged inner mitochondrial membrane. These nanoparticles self-assemble *via* disulfide bonds, which are selectively cleaved by mGSH. Upon cleavage, Ir^3+^ complexes are released, and simultaneously lowering endogenous GSH levels. Under two-photon laser irradiation, both the nanoparticles and released Ir^3+^ generate a mixture of ^1^O_2_ and •O_2_^−^, thereby amplifying intracellular oxidative stress. The depletion of GSH combined with increased ROS production inhibits the biosynthesis of glutathione peroxidase 4 (GPX4), a key lipid repair enzyme (Zhang Y. et al., 2021). Downregulation of GPX4 is widely recognized as a hallmark of ferroptosis (Zou et al., 2024a). This study demonstrates that IrS NPs precisely trigger ferroptosis by reducing mitochondrial GSH levels and promoting ROS generation. This work offers a novel strategy for enhancing PDT through organelle-targeted approaches, potentially overcoming limitations associated with conventional apoptosis-inducing therapies and holding significant clinical potential.

Besides the direct depletion of intracellular GSH by disulfide bonds, delivering carbon monoxide (CO) has also been shown to enhance the therapeutic effect of PDT by suppressing mitochondrial biosynthesis. High doses of CO can induce apoptosis in cancer cells by inhibiting mitochondrial respiration and interfering with protein synthesis (Dong et al., 2024; Yu et al., 2024c). Porphyrin-based nMOF materials have become a major focus in phototherapy research in recent years (Zhang et al., 2024; Zou et al., 2024b; Zou et al., 2025a). However, achieving more precise treatment by targeting specific intracellular organelles may serve as a powerful driving force for further advancing the biomedical applications of nMOFs. Yang *et al.* designed a multi-layered targeting nanosystem (HA@MR@PCN-CORM) with hierarchical targeting strategies (Figure 3B), in which CORM-40-a transition metal carbonyl compound capable of controlled CO release-was loaded into the hollow structure of porphyrin-based PCN-224 (Yang F. et al., 2023). By surface coating with hyaluronic acid (HA), a natural polysaccharide that binds to CD44 receptors overexpressed on tumor cells, the nanosystem could realize active tumor targeting. Moreover, incorporation of a mitochondria-targeted ROS-responsive amphiphilic copolymer (MR), which contains triphenylphosphonium (TPP) groups, it could accumulate in mitochondria. The TPP groups are initially shielded by HA, preventing premature mitochondrial targeting in normal tissues. Upon HA degradation by tumor-associated hyaluronidase, TPP is exposed to mediate secondary mitochondrial targeting (Figure 3C). The core of the system is PCN-224, a porphyrin-based MOF loaded with CORM-40, a CO-releasing molecule. Under 660 nm laser irradiation, PCN-224 generates ROS, which cleaves the thioketal linker in MR, triggering controlled CO release. CO then downregulates glutamate-cysteine ligase and glutathione synthetase in 4T1 cells, inhibiting mGSH synthesis. In xenograft tumor models, HA@MR@PCN-CORM treatment led to significantly reduced levels of GPX4 and glutamate-cysteine ligase proteins, further confirming that mitochondrial CO release can sensitize cancer cells to ferroptosis through GSH suppression, thereby enhancing apoptosis and achieving effective antitumor activity.

### 3.2 Direct O_2_ generation in mitochondria alleviates hypoxia and enhances PDT efficacy

Hypoxic tumor microenvironment remains a major obstacle limiting the efficacy of O_2_-dependent PDT in suppressing cancer cell proliferation (Shen et al., 2022; Xie et al., 2024). Therefore, direct strategies to generate O_2_ within tumor cells can significantly enhance PDT outcomes (Zhang et al., 2022a). Mitochondria are both major O_2_ consumers *via* oxidative phosphorylation and key PDT targets, therefore, *in situ* O_2_ generation within mitochondria can simultaneously replenish O_2_ for ^1^O_2_ production and protect this organelle from hypoxic damage. The mitochondrial matrix’s unique microenvironment, such as high H_2_O_2_ levels and acidic pH, makes it an ideal site for catalytic O_2_ generation. Numerous approaches have been developed, such as using inorganic catalysts like Mn_3_O_4_ and Pt nanoparticles, which react with endogenous H_2_O_2_ to produce O_2_ (Xu et al., 2018; Zhao et al., 2023). Alternatively, catalase enzymes can be employed to directly convert H_2_O_2_ into O_2_ (Cheng et al., 2020; Sun H. et al., 2023).

**FIGURE 4:**
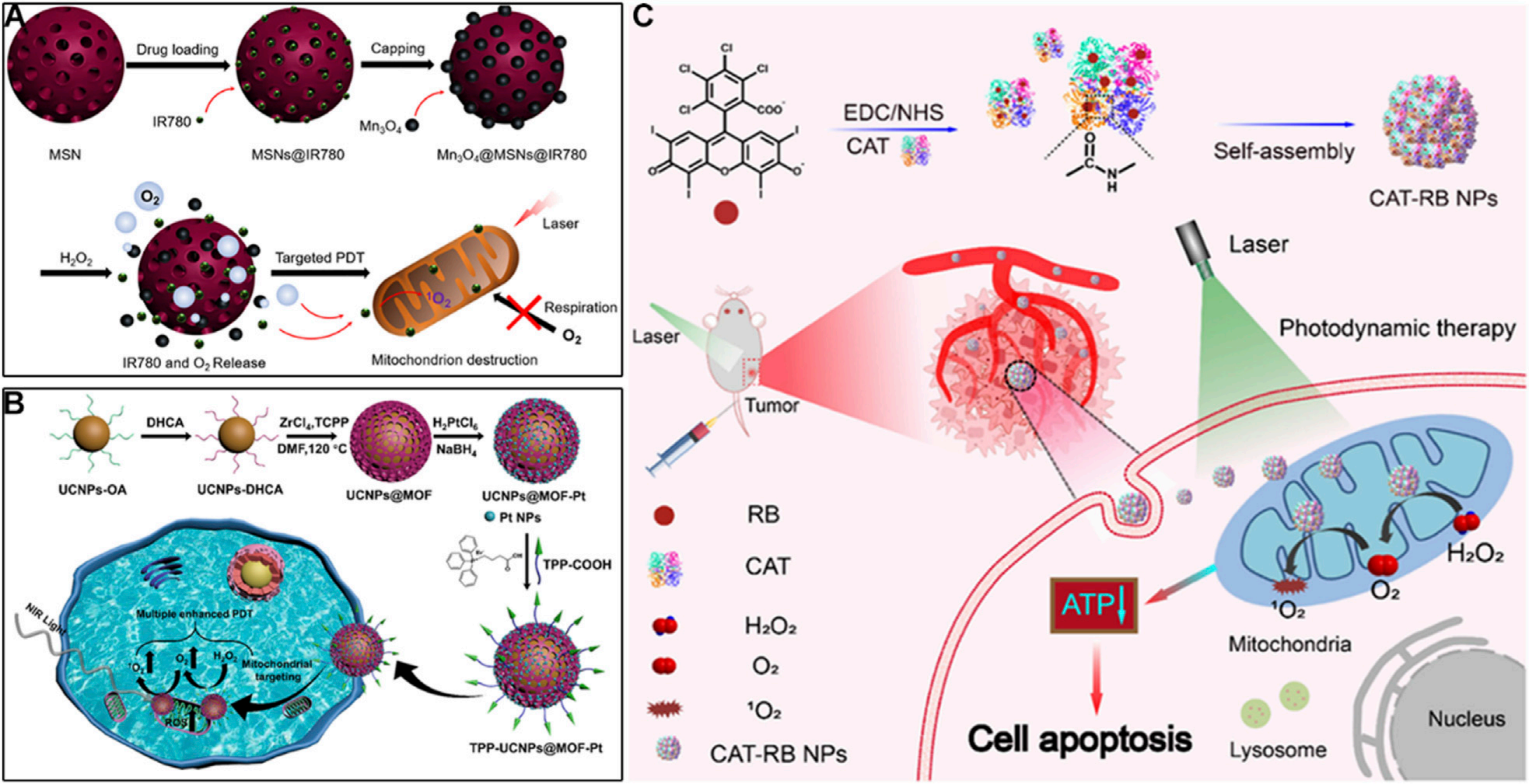
**(A)** Preparation of the Mn_3_O_4_@MSN@IR780 nanoplatform with mitochondrial targeting *via* IR780 and pH-responsive O_2_-generating capability, along with its mechanism for enhanced PDT. Reproduced with permission from Ref. Yang et al. (2019). Copyright ^©^ 2019 Ivyspring International Publisher. **(B)** Preparation process of the TPP-UCNP@MOF-Pt nanoplatform featuring TPP-mediated mitochondrial targeting and Pt-catalyzed O_2_ production, and its mechanism in boosting PDT. Reproduced with permission from Ref. Chen et al. (2023c). Copyright ^©^ 2023 American Chemical Society. **(C)** Schematic illustration of the CAT-RB NP system with cardiolipin-mediated mitochondrial targeting for catalase-driven O_2_ generation and augmented ^1^O_2_ production in PDT. Reproduced with permission from Ref. Xu et al. (2023b). Copyright ^©^ 2023 Chinese Chemical Society.

For example, Guan *et al.* developed mesoporous silica nanoparticles co-loaded with lipophilic cation IR780 and Mn_3_O_4_ nanoparticles (Figure 4A) (Yang et al., 2019). IR780, a lipophilic cationic cyanine dye, serves as the mitochondrial targeting ligand. IR780’s positive charge drives accumulation in mitochondria, while its hydrophobic structure enables insertion into the inner mitochondrial membrane. The mesoporous silica core acts as a carrier for Mn_3_O_4_ nanoparticles, protecting them from premature degradation and ensuring co-delivery with IR780 to mitochondria. With pH-dependent activity, Mn_3_O_4_ reacts with H_2_O_2_ to generate O_2_ in mitochondrial matrix-mimicking conditions. This O_2_ supply strategy enhances IR780-mediated ^1^O_2_ generation, while PDT-induced mitochondrial damage reduces O_2_ consumption, creating a “self-amplifying” effect. Zhu *et al.* fabricated a hybrid nanoplatform by combining upconversion nanoparticles (UCNPs) with MOFs, and decorated the surface with platinum nanoparticles and mitochondria-targeting TPP groups (Figure 4B) (Chen Y. et al., 2023). In this system, Pt efficiently catalyzes O_2_ generation from H_2_O_2_, alleviating tumor hypoxia and boosting PDT. Additionally, mitochondrial targeting leads to membrane depolarization and activation of apoptotic pathways, further enhancing therapeutic performance.

Beyond inorganic catalysts, enzymatic O_2_ generation offers higher specificity, as catalase directly converts H_2_O_2_ to O_2_ with high efficiency. Xu *et al.* catalase with rose bengal (RB) to form CAT-RB nanoparticles, where RB acts as both a photosensitizer and a mitochondrial targeting moiety *vi*a its high affinity for cardiolipin, a unique phospholipid enriched in the inner mitochondrial membrane (Figure 4C) (Xu X. et al., 2023). This cardiolipin-RB interaction ensures higher mitochondrial colocalization far exceeding cytosolic distribution. The covalent conjugation of catalase to RB through a PEG linker ensures that O_2_ generated by catalase is locally retained in mitochondria, maximizing its efficacy for ^1^O_2_ production. After cellular uptake, CAT-RB specifically accumulate in mitochondria, maintaining high catalase activity to converts H_2_O_2_ into O_2_, and increasing ^1^O_2_ production during PDT under 546 nm irradiation. The generated ROS further damage mitochondria and reduce ATP levels, promoting cancer cell apoptosis. These O_2_-generating strategies are straightforward and effective, offering promising solutions to overcome hypoxia-associated limitations in PDT and significantly improving its anticancer potential.

### 3.3 Reduce mitochondrial respiration in mitochondria for enhanced PDT

Mitochondria is a crucial organelle targeted by PDT, and excessive ROS accumulation can initiate the mitochondrial apoptosis pathway, resulting in cell death (Zuo et al., 2021). Positioning fundamental elements of PDT into mitochondria has received considerable attention for improving ROS transportation, thus realizing PDT with high specificity (Li et al., 2021). Due to the high consumption of O_2_ caused by mitochondrial respiration, sabotaging mitochondria and eliminating mitochondrial respiration are expected effective strategies to amplify the antitumor efficacy of PDT (Guo et al., 2021; Hu et al., 2024). Inhibiting respiration reduces O_2_ consumption, increasing local O_2_ availability for Type II PDT, while simultaneously sensitizing cells to ROS by disrupting mitochondrial membrane potential and reducing ATP-dependent repair mechanisms.

**FIGURE 5:**
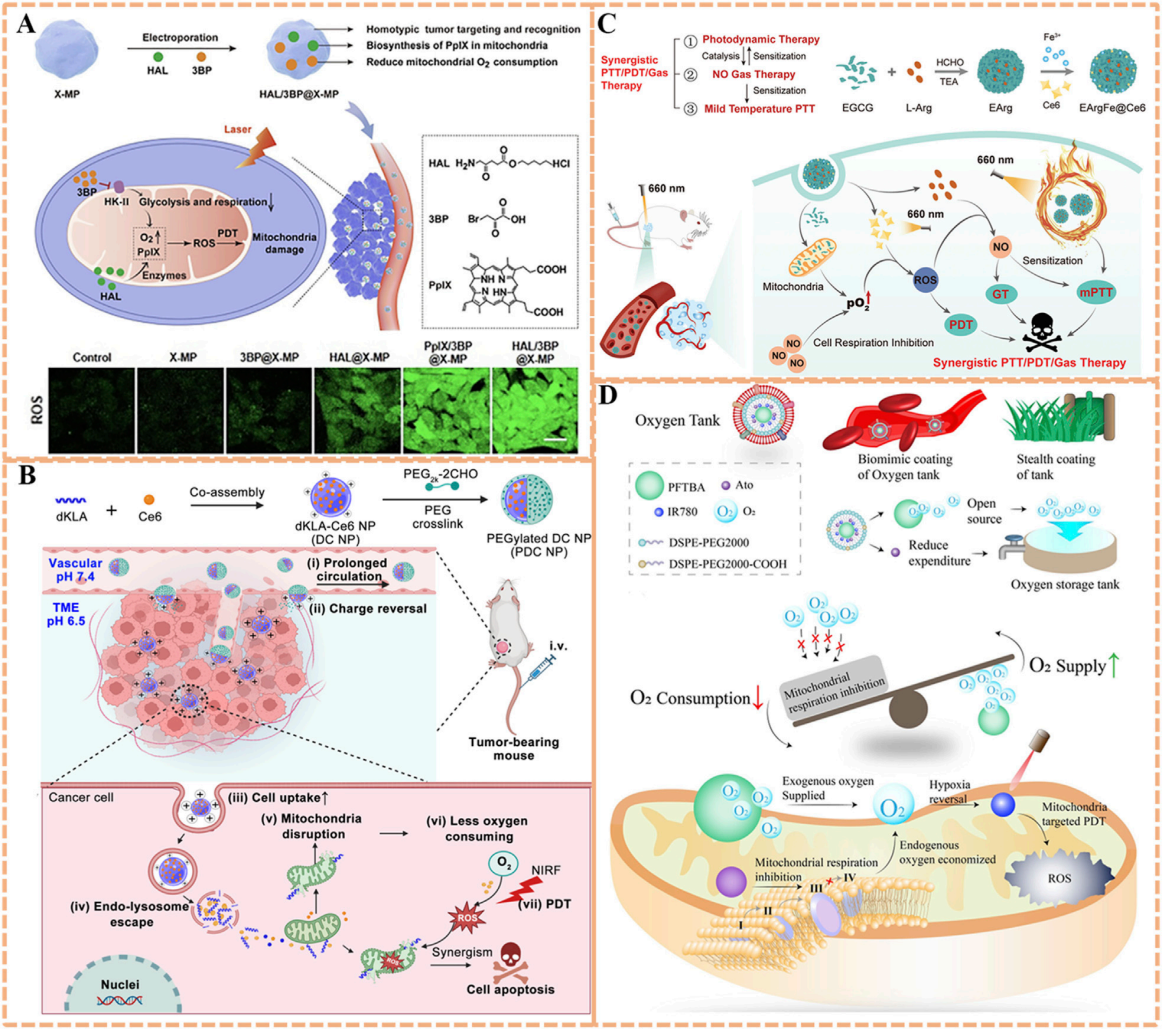
**(A)** Preparation of the HAL/3BP@X-MP nanoplatform with homotypic tumor targeting and HK-II-inhibiting capability, along with its mechanism for potent PDT via O_2_ conservation. Reproduced with permission from Ref. Zuo et al. (2021). Copyright ^©^ 2021 WILEY-VCH Verlag GmbH and Co. KGaA. **(B)** Preparation process of the PDC NP system featuring pH-responsive tumor accumulation and dKLA-mediated mitochondrial targeting, and its mechanism in synergistic PDT/peptide therapy. Reproduced with permission from Ref. Qu et al. (2023). Copyright ^©^ 2023 Springer-Verlag. **(C)** Schematic illustration of the EArgFe@Ce6 nanoplatform with EGCG-Fe^3+^-mediated mitochondrial targeting for combined PDT/PTT/gas therapy via respiration inhibition. Reproduced with permission from Ref. Shi et al. (2023). Copyright ^©^ 2023 WILEY-VCH Verlag GmbH and Co. KGaA. **(D)** Preparation of the O_2_ Tank nanoplatform with RBCm-enabled prolonged circulation and dual-mode hypoxia alleviation, along with its mechanism for enhanced mitochondrial PDT. Reproduced with permission from Ref. Li et al. (2022b). Copyright ^©^ 2022 BioMed Central.

To enhance the accumulation of O_2_ and photosensitizers within mitochondria precisely, hexyl 5-aminolevulinate hydrochloride (HAL) and 3-bromopyruvic acid (3BP) were simultaneously encapsulated into microparticles of X-ray irradiated tumor cells (X-MP) to prepareHAL/3BP@X-MP for potent PDT (Figure 5A) (Zuo et al., 2021). Leveraging homotypic targeting, X-MP retains tumor cell surface antigens (*e.g.*, integrins, E-cadherin), enabling specific recognition and uptake by homologous tumor cells. The payload 3BP could serve as a small-molecule inhibitor target to hexokinase-II (HK-II), an enzyme that binds to the outer mitochondrial membrane *via* voltage-dependent anion channel interactions, linking glycolysis to mitochondrial respiration. 3BP covalently modifies HK-II’s active site, significantly increasing local O_2_ supply by inhibiting mitochondrial respiration and glycolysis. Concurrently, HAL promotes the localized biosynthesis and effective accumulation of protoporphyrin IX (PpIX) in mitochondria *via* the heme biosynthesis pathway, resulting in high PpIX accumulation in mitochondria. Additionally, PpIX can produce sufficient ROS to directly destroy the mitochondria of cancer cells, leading to outstanding antitumor effects of PDT. In both *in vitro/vivo* experiments, the remarkable antitumor capability of HAL/3BP@X-MP could be observed without obvious side effects, providing a prospect for conquering the limitations of the PDT modality to fight hypoxic tumors.

Peptide-based respiration inhibitors could also offer high specificity. d-(KLAKLAK)_2_ (dKLA), a 14-amino-acid α-helical proapoptotic peptide, has been demonstrated to damage mitochondria of eukaryotic cells, abrogate mitochondria respiration, reduce O_2_ consumption (Qu et al., 2023). Accordingly, dKLA is conductive to improve the photodynamic therapeutic effect when combined with photosensitizers. Inspired by this, a charge-reversible crosslinked nanoparticle (PDC NP) based on photosensitizer chlorin e6 (Ce6) and dKLA was developed for strengthened peptide delivery efficiency and synergistic antitumor effect (Qu et al., 2023). As illustrated in Figure 5B, the proapoptotic dKLA was co-assembled with Ce6 to construct pure drug nanoparticles (DC NP) with drug loading capacity of 100%. Subsequently, a dual-aldehyde terminated PEG_2k_-2CHO was utilized to crosslink the DC NP to obtain the surface charge reversal PEGylated DC NPs (PDC NP, 68.3% drug loading). Crosslinking with a dual-aldehyde PEG, theses pH-responsive NPs confer a slightly negative surface charge in blood for prolonged circulation, and undergoes charge reversal to positive in acidic tumor microenvironments, promoting cellular internalization. dKLA’s targeting mechanism relies on its cationic α-helical structure, which interacts with anionic phospholipids in the outer mitochondrial membrane and inserts into the membrane, causing depolarization. The dKLA could target and disrupt the mitochondrial membrane, and next inhibit the mitochondrial respiratory chain to reduce the consumption of local O_2_. In addition, the concentration of Ce6 around mitochondria would increase in company with the targeting delivery of dKLA, thus extensively augmenting ^1^O_2_ production mediated by Ce6 due to increased O_2_ availability. This strategy may offer a valuable approach for magnifying the drug delivery capability of peptides and optimizing mitochondrial-targeted PDT efficiency.

Apart from the well-known effect on tumor cell-killing directly, the antitumor effect induced by nitric oxide (NO) involves multiple pathways, such as nitrosation of mitochondria and DNA, as well as inhibition of cellular respiration, which is effective for overcoming drug resistance in PDT (Xu et al., 2021; Jiang et al., 2022). A mitochondria-targeted NO nanogenerator termed EArgFe@Ce6 was developed to achieve synergistic photodynamic/gas/photothermal tumor therapy (Figure 5C) (Shi et al., 2023). EGCG-Arg nanoparticles (EArg) were formed *via* chemical assembly of epigallocatechin gallate (EGCG) and NO donor l-Arginine (l-Arg), then EArgFe was prepared by adding ferric ions (Fe^3+^) into EArg to enable the coordination between Fe^3+^ and EGCG, and finally Ce6 was physically decorated on its surface to construct EArgFe@Ce6 (Figure 8B). Upon 660 nm light irradiation, the coordination of EGCG and Fe^3+^ endows nanogenerator with prominent photothermal capability for low-temperature PTT (Figure 8C). EGCG has verified hypoxia alleviation by inhibiting mitochondrial respiration, therefore EArgFe@Ce6 could target to and accumulate in mitochondria to suppress cell respiration, favoring the improvement of PDT. The ample ROS produced by PDT would in-turn catalyze l-Arg to synthesize considerable NO for gas therapy. The mitochondrial localized NO can suppress cell respiration and further sensitize PDT and low-temperature PTT. Together, the nanogenerator has been manifested with superior photodynamic/gas/photothermal therapeutic outcomes in *in vitro* and *in vivo* experiments, which almost achieve complete tumor ablation.

Off-target respiratory inhibition remains a challenge, as systemic delivery can affect normal tissues (*e.g.*, heart, brain) with high O_2_ demand (Xu et al., 2022; Zeng et al., 2024a). Li *et al.* addressed this with an “O_2_ Tank” system: red blood cell membrane (RBCm)-coated perfluorocarbon (PFC) liposomes co-loaded with atovaquone (ATO, a Complex III inhibitor) and IR780 (Figure 5D) (Li X. et al., 2022). By a biomimetic stealth strategy, RBCm coating provides immune evasion and prolonged circulation, while PFC’s high O_2_ solubility acts as an exogenous O_2_ reservoir. Essentially, ATO, a lipophilic drug, passively accumulates in mitochondria via partitioning into lipid membranes, where it selectively inhibits Complex III of the respiratory chain, reducing tumor O_2_ depletion without affecting normal tissues. IR780, as a mitochondrial-targeted photosensitizer, utilizes both endogenous (respiration-inhibited) and exogenous (PFC-delivered) O_2_ to generate ^1^O_2_, thus boosting PDT for amplified tumor growth inhibition. This current strategy concentrates on synergistic tumor hypoxia modulation *via* exogenous O_2_ supply and inherent O_2_ metabolism, exerting the potential to augment outcomes of various therapies with hypoxia-associated resistance.

### 3.4 PDT with O_2_-independent pathways

The therapeutic effect of photosensitizers in PDT largely depends on the ROS production efficiency and tissue penetration depth, therefore, type II PDT which relies on O_2_ to produce ^1^O_2_ always displays insufficient effect in deep hypoxic region (Zhuang et al., 2025). In contrast, Type I PDT circumvents O_2_ dependence by generating cytotoxic radicals (*e.g.*, •O_2_^−^, •OH) *via* electron/proton transfer reactions with endogenous donors (*e.g.*, NADH, GSH), maintaining ROS production even when O_2_ is depleted (Zheng X. et al., 2021). This O_2_ independence makes Type I photosensitizers particularly valuable for treating hypoxic tumors, where they can exploit the highly reducing mitochondrial matrix (rich in redox-active molecules) to amplify radical generation (Zhuang et al., 2023).

**FIGURE 6:**
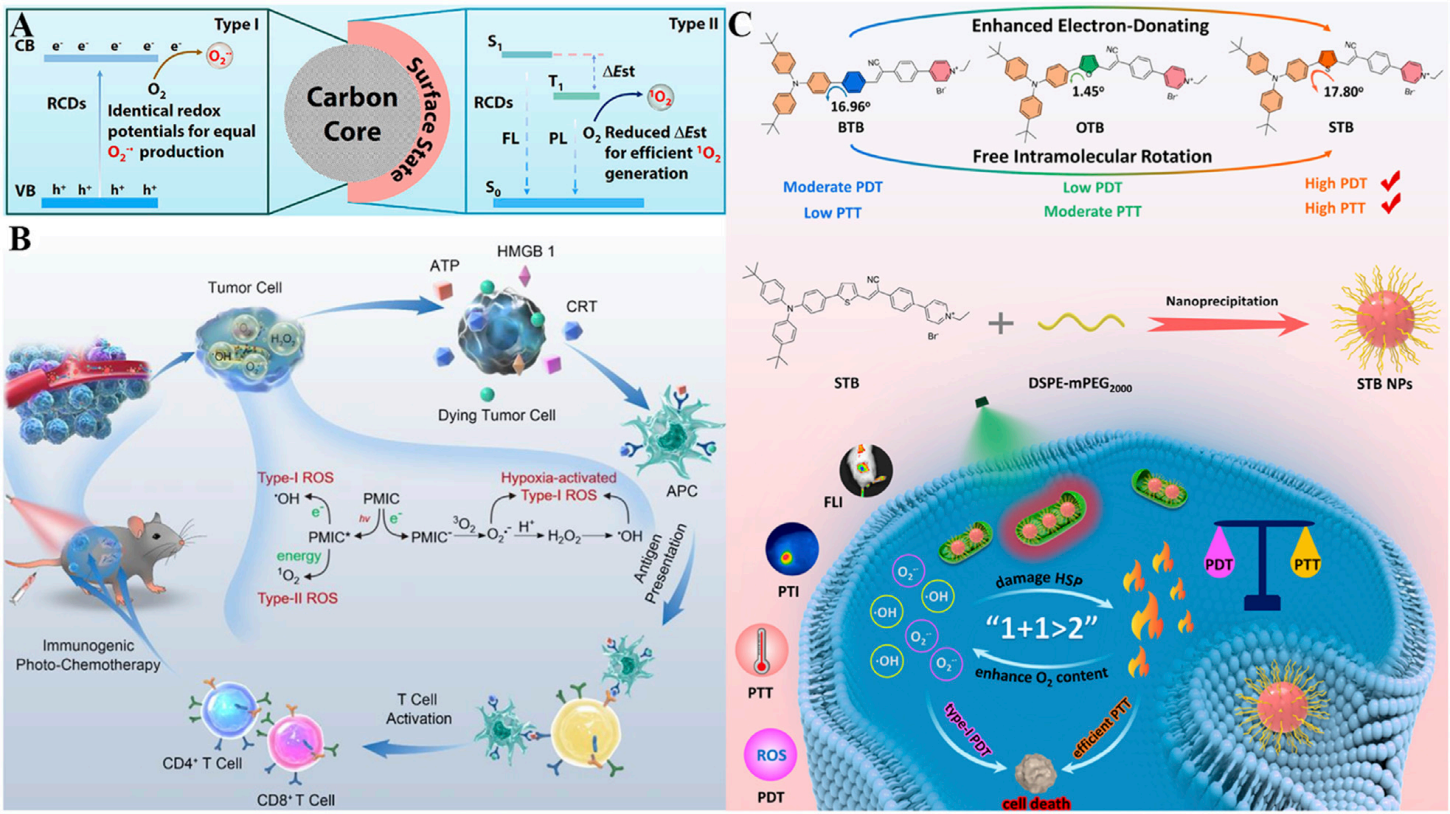
**(A)** Mechanism of the RCD nanoplatform with tunable Type I/II ROS production capability *via* surface state regulation, along with its dual-mode PDT mechanism. Reproduced with permission from Ref. (Zhang et al., 2023d). Copyright ^©^ 2023 Elsevier Ltd. **(B)** Schematic of the PMIC-NC system featuring NIR light-activated Type I/II ROS generation, hypoxia-triggered Type I ROS burst, and ICD induction for synergistic antitumor therapy. Reproduced with permission from Ref. Lou et al. (2023). Copyright ^©^ 2023 WILEY-VCH Verlag GmbH and Co. KGaA. **(C)** Preparation of π bridge-engineered STB phototheranostic agents with enhanced Type I PDT and PTT performance, along with their mechanism for synergistic therapy. Reproduced with permission from Ref. Fang et al. (2023a). Copyright ^©^ 2023 American Chemical Society.

To achieve a satisfactory PDT effect in aerobic and hypoxic scenarios simultaneously, three types of red light-emissive CDs (RCDs) derived from *hypericum perforatum* extract with adjustable type I/II ROS production have been developed (Li M. et al., 2025). These RCDs exhibit intrinsic mitochondrial targeting *via* surface amine groups, which interact electrostatically with the negatively charged inner mitochondrial membrane, achieving high mitochondrial colocalization. Their unique structural design enables dual-mode ROS generation, the RCDs exhibit tunable ROS generation with equal •O_2_^−^ *via* type I PDT and incremental ^1^O_2_ *via* type II PDT, which could be adopted for deep red fluorescence imaging-guided type I/II PDT. On one hand, the unaltered core sizes of RCDs donated their same redox potentials, resulting in the production of equal •O_2_^−^ (Figure 6A) (Zhang et al., 2023d). On the other hand, the enhancement of ^1^O_2_ production was demonstrated to be ascribed to their surface states, thus reducing the energy gaps between S_1_ to T_1_. With inherent mitochondria targeting ability, RCDs could precision attack towards tumors. *In vitro* and *in vivo* studies demonstrated RCDs with tunable type I/II ROS production capability, realizing a remarkable anticancer eradication even under harsh hypoxic tumor microenvironment. This work offers considerable prospects for developing versatile CDs as photosensitizers with adjustable ROS production to overcome the limitation of current single-type PDT, exhibiting substantial potential in antitumor applications.

Immunotherapy has been widely studied for cancer treatment in recent years (Chen and Wang, 2020; Hu et al., 2020; Song et al., 2022; Mao et al., 2023; Zhao et al., 2024). The combination of PDT with immunotherapy offers a promising new direction for the treatment of malignant cancer cells (Ji et al., 2022; Liu et al., 2025). Aside from directly inducing tumor cell death by PDT, ROS can further induce ICD effect for efficient tumor ablation (Huang et al., 2022; Wei et al., 2022). To improve the ROS-induced ICD effect, one soluble perylene monoimide derivative bearing tertiary ammonium functions at the imide and peri positions (PMIC-NC) was synthesized for augmented PDT therapy. Serving as a ROS supergenerator, the prepared PMIC-NC could induce Type-I/II ROS generation through electron/energy transfer upon 660 nm laser irradiation as well as trigger endogenous burst of Type I ROS assisted by proton transients in tumor cells. The excessive ROS could directly kill cells by PDT on the primary tumor, furthermore elicit vigorous ICD effect to awake antitumor immunity (cytokine secretion, DCs maturation, cytotoxic T lymphocytes activation) and thereby curb the proliferation of distant or metastasis tumors (Figure 6B) (Lou et al., 2023). After therapies, PMIC-NC supplied preferable antitumor effects on both primary tumors (suppression rate of 85.4%) and pulmonary metastasis (suppression rate of 58.1%) upon NIR irradiation. This work thus provides one proof-of-concept for the design of ROS generators which incorporates NIR light-activated Type-I/II PDT, hypoxia-triggered Type-I ROS burst and ICD effect, to realize immunogenic photochemotherapy towards hypoxic tumors.

Synergizing PDT with PTT leverages photon energy for both ROS generation and hyperthermia, with mitochondria as a critical co-target due to their sensitivity to thermal damage (Cui et al., 2023). Given that the combinational therapy of PDT and PTT may maximize treatment outcomes, Fang *et al.* have synthesized three mitochondria-targeting phototheranostic agents for multimodal imaging-guided PDT/PTT synergistic therapy (Figure 6C) (Fang L. et al., 2023). Attributed to the cationic pyridinium moiety, (Z)-4-(4-(2-(5-(4-(bis(4-(tert-butyl)phenyl)amino)phenyl)thiophen-2-yl)-1-cyanovinyl)phenyl)-1-ethylpyridin-1-ium bromide (STB) shows mitochondria-targeting ability *via* electrostatic interactions with the inner mitochondrial membrane. Employing thiophene as π bridge, STB strengthens robust donor-accepter (D-A) interactions, separates the distribution of HOMO and LUMO, enabling it with prominent NIR fluorescence emission. Moreover, the decrease of the S_1_-T_1_ energy gap facilitates the intersystem crossing (ISC), allowing free intramolecular rotation and resulting in massive amounts of •O_2_^−^ and •OH production by optimal type I PDT. In the meantime, an ideal molar extinction coefficient and adequate intramolecular motions are beneficial for the enhancement of photothermal conversion efficiency (up to 51.9%). Based on these, STB NPs could actively target to and accumulate in the tumor site, realize real-time *in vivo* NIR fluorescence imaging, and obviously inhibit tumor growth with negligible side effects. This work subtly established a practical strategy by π bridge engineering, for designing phototheranostic agents with the enhancement of PDT/PTT performance, accelerating the progress of multimodal imaging-guided phototherapy against tumors.

**FIGURE 7:**
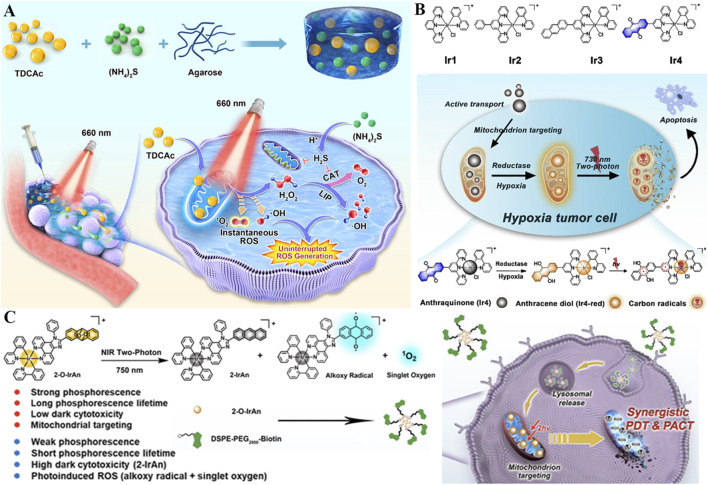
**(A)** Schematic of the TSH hydrogel-based uninterrupted ROS generator with H_2_S-mediated catalase inhibition, along with its mechanism for combined H_2_S gas therapy and Type I PDT. Reproduced with permission from Ref. Zhang et al. (2022c). Copyright ^©^ 2022 Elsevier Ltd. **(B)** Mechanism of the Ir4 system with hypoxia-activated two-photon excitation and mitochondrial carbon radical generation for PDT in hypoxic tumor cells. Reproduced with permission from Ref. Kuang et al. (2020). Copyright ^©^ 2020 WILEY-VCH Verlag GmbH and Co. KGaA. **(C)** Illustration of the Ir(III) complex-based system featuring synergistic PDT and photoactivated chemotherapy *via* NIR-triggered ROS release and cytotoxic precursor activation. Reproduced with permission from Ref. Kuang et al. (2022). Copyright ^©^ 2022 American Chemical Society.

Despite their hypoxia resistance, Type I photosensitizers face challenges: short radical lifetimes and limited intratumoral accumulation (Xiong et al., 2025). To address this, an injectable hydrogel (TSH) was engineered to co-deliver TDCAc (a Type I photosensitizer) and (NH_4_)_2_S (a donor of H_2_S), aiming at sustained ROS generation (Figure 7A) (Zhang T. et al., 2022). Upon 660 nm laser irradiation, TDCAc would rapidly gain heat due to PTT effect, promoting the dissolution of agarose hydrogel, accelerating the release of (NH_4_)_2_S and TDCAc in the tumor. Afterward, H_2_S gas was generated by (NH_4_)_2_S in an acidic tumor microenvironment, diffused into tumor cells, and then repressed intracellular catalase activity. Due to reduced H_2_O_2_ consumption, the in-turn accumulation of H_2_O_2_ could induce continuous •OH generation *via* the Fenton reaction by utilizing the endogenous labile iron pool in cancer cells. Notably, H_2_S also upregulates mitochondrial Fe^2+^ transporters (*e.g.*, DMT1), amplifying Fenton reactions in a feedforward loop and increasing •OH levels. The uninterrupted ROS production in cancer cells and distinguished tumor ablation have been demonstrated *in vitro* and *in vivo*. In recent years, drug delivery research based on hydrogel carriers has emerged as a promising field (Xia et al., 2023; Xu L. et al., 2023; Zhang H. et al., 2023). Combining hydrogels with phototherapy may offer new insights and strategies for the treatment of malignant cancers (Chen et al., 2021).

Two-photon excited PDT (2PE-PDT) utilizes a photosensitizer with short wavelength absorption to absorb two NIR photons and subsequently trigger ROS production, which offers an enormous possibility for precise treatment against deep tumor tissues (Wu et al., 2021; Tan et al., 2025). In this context, a series of iridium (III) complexes (Ir1-4) were synthesized, and anthraquinone was inserted in Ir4 as a mitochondrion-localized carbon radical initiator and an emission quenching group (Figure 7B) (Kuang et al., 2020). Under hypoxic conditions, this anthraquinone group within Ir4 could be specifically reduced by reductase and formed anthracene diol (Ir4-red), thus turning on the two-photon excited capability of complexes. Subsequent irradiation results in mitochondrion-localized carbon radical production, excessive intracellular ROS accumulation, and damage to mitochondrial membrane potential, resulting in cell death (IC_50_^light^ = 2.1 μM). Due to the hypoxia-activated and mitochondrion-targeted merits of two-photon excitation, Ir4 has demonstrated dual functions of two-photon excited imaging (lex = 730 nm) and remarkably PDT therapy *in vivo* against hypoxic conditions. This metal complex-based two-photon photosensitizer could generate carbon radicals in an O_2_-independent way, which is promising as a candidate for clinical treatment of hypoxic tumors.

On this basis, Kuang *et al.* reported a novel photoactivated prodrug (2-O-IrAn) based on the iridium (III) endoperoxide complex, which effectively united the characteristics of two-photon photosensitizers and photoactivated chemotherapy prodrugs with low dark toxicity. 2-O-IrAn targets mitochondria *via* its lipophilic cationic structure, achieving high colocalization with mitochondria, and is activated by NIR two-photon irradiation to release ^1^O_2_, alkoxy radicals, and cytotoxic precursor 2-IrAn (a mitochondrial Complex I inhibitor) (Figure 7C) (Kuang et al., 2022). This photoactivation reaction can disrupt the mitochondrial membrane and ultimately cause tumor cell death efficiently under both normal O_2_ (PI = 959.1) and anaerobic conditions (PI = 690.3), appreciably enhancing the therapeutic effect of hypoxic PDT and photoactivated chemotherapy. In addition, DSPE-PEG-Biotin was introduced to endow DSPE-PEG-Biotin@2-O-IrAn nanoparticles with excellent tumor targeting and prolonged blood half-life, which could realize complete tumor eradication *in vivo*. This synergistic strategy of NIR two-photon photoactivated chemotherapy and PDT provides a novel guideline for the treatment of hypoxic tumors.

However, mitochondria-targeted PDT is also constrained by multi-dimensional limitations: In terms of targeting specificity, membrane potential-dependent carriers exhibit reduced localization efficiency in hypoxic tumor cells due to decreased mitochondrial membrane potential, and may be affected by variations in tumor metabolic phenotypes. At the level of carriers, metal-based nanoparticles (*e.g.*, Pt, Ir) may interfere with mitochondrial iron-sulfur cluster synthesis to trigger chronic toxicity, peptide drugs are susceptible to protease degradation, and enzymatic catalysts show poor stability in acidic tumor microenvironments. In terms of therapeutic mechanisms, mGSH depletion may trigger compensatory elevation of cytosolic GSH, short-lived radicals tend to induce Nrf2-mediated antioxidant responses, and respiratory inhibitors may carry the risk of off-target toxicity. Additionally, excess inhibition of mitochondrial respiration may activate protective autophagy, thereby weakening therapeutic effects. These challenges collectively restrict the clinical translation and efficacy optimization of mitochondria-targeted PDT.

## 4 Targeting lysosome for enhanced PDT

Lysosomes are organelles that contain large amounts of cathepsin to perform key cellular catabolic processes, which are essential for maintaining cell homeostasis and regulating various cellular processes (Zhang Z. et al., 2021; Gros and Muller, 2023). Different from the normal cells, lysosomes in tumor cells are more plentiful with altered morphology, which upregulated lysosomal activity in tumor tissue is related to the metabolic need of fast proliferating tumors (He et al., 2023; Jia et al., 2023). In tumor tissue, photosensitizers localized in the lysosome may cause selective damage to lysosomes and induce subsequent cathepsin release into the cytoplasm, trigger cell death, and finally postpone the progression and metastasis of tumors.

**FIGURE 8:**
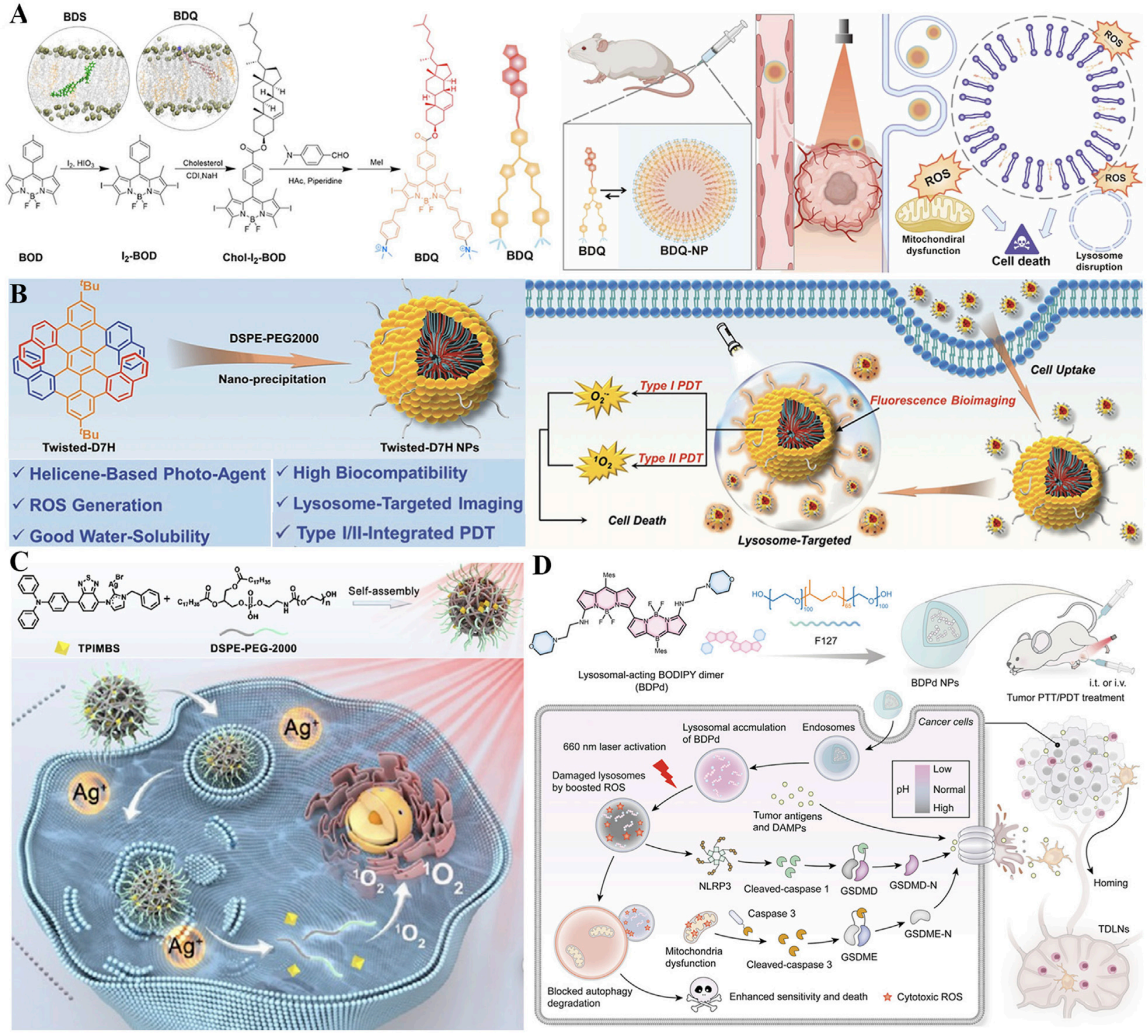
**(A)** Preparation of the BDQ-NP nanoplatform with pH-dependent lysosomal targeting via protonated amino groups and ROS-mediated lipid peroxidation capability, along with its mechanism for enhanced PDT and metastasis inhibition. Reproduced with permission from Ref. Li et al. (2023). Copyright ^©^ 2023 Royal Society of Chemistry. **(B)** Preparation process of the Twisted-D7H NP system featuring DSPE-PEG2000-enabled water solubility and lysosome-specific accumulation, and its mechanism in Type I/II PDT. Reproduced with permission from Ref. Zhao et al. (2022). Copyright ^©^ 2022 WILEY-VCH Verlag GmbH and Co. KGaA. **(C)** Preparation of the TPIMBS NP nanoplatform with lysosomal targeting and photoactivable Ag^+^ release capability, along with its mechanism for synergistic PDT/chemotherapy and prolonged bioimaging. Reproduced with permission from Ref. Liu et al. (2023a). Copyright ^©^ 2022 American Chemical Society. **(D)** Preparation process of BDPd NP system featuring morpholine-mediated lysosomal targeting and NIR-responsive dual PDT/PTT functionality, and its mechanism in synergistic therapy. Reproduced with permission from Ref. Sun et al. (2024). Copyright ^©^ 2023 American Chemical Society.

The alteration of lysosomal membrane permeabilization (LMP) can disrupt the function of lysosomes and even cause lysosome-dependent cell death (Tanaka et al., 2022). By inhibiting the leakage of photosensitizers from lysosomes, constant LMP and unrecoverable damage to cancer cells could be motived under light irradiation, thus the efficiency of PDT substantially improved. Encourage by this, Li *et al.* reported lysosome-targeting nanophotosensitizer (BDQ-NP) based on boron-dipyrromethene (BOD) derivatives with an amphiphilic structure (synthesized *via* a reaction involving BOD core modification and hydrophobic chain conjugation) for highly effective PDT (Figure 8A) (Li et al., 2023). Amphiphilic BDQ could be synthesized in four steps by BOD and subsequently self-assembled into nano-sized particles (BDQ-NP) in aqueous solution at a concentration of 0.5 mg/mL under mild stirring (200 rpm) for 2 h. Owing to the pH-dependent lysosome-targeting property (facilitated by protonation of amino groups in the acidic lysosomal environment), BDQ powerfully incorporates into the lipid bilayers of lysosomes and induces continuous LMP through ROS-mediated LPO. Under light irradiation (660 nm, 10 mW/cm^2^), BDQ-NP produced a substantial amount of ROS, and disrupted functions of lysosome and mitochondria *via* activating the cathepsin B-mediated mitochondrial damage pathway. Specifically, cathepsin B cleaves pro-apoptotic proteins to trigger outer membrane permeabilization, releasing cytochrome c and initiating caspase-dependent apoptosis. After intravenous injection, BDQ-NP achieved high accumulation in tumors as well as a fantastic PDT effect on subcutaneous colorectal CT26 tumors and orthotopic 4T1 breast carcinomas with low systemic toxicity. In addition, PDT mediated by BDQ-NP also efficiently hindered lung metastasis of 4T1 breast carcinomas by reducing MMP-9 expression (downregulated by 42%) *via* lysosomal damage. This work highlights the potential value of self-assembled lysosome-targeted photosensitizers for augmented PDT effect against tumor metastasis.

The characteristics of inherent chirality, nonplanarity, and structural controllability make helicenes promising candidates in the field of optoelectronics and biomedical applications (Bouz et al., 2025). Nevertheless, the poor water solubility of helicenes strictly restricts their biological applications for organelle-targeted type I and II PDT (Yang et al., 2024). To overcome these limitations, water-soluble nanoparticles named D7H-NPs comprising twisted double [7]carbohelicene (D7H) were fabricated for lysosome-targeted bioimaging and advanced PDT (Zhao et al., 2022). As shown in Figure 8B, D7H was reprecipitated with amphiphilic 1,2-distearoyl-sn-glycero-3-phosphoethanolamine N-[methoxy (polyethylene glycol)-2000] (DSPE-PEG2000) to prepare self-assembled D7H-NPs *via* the nanoprecipitation method (Zhao et al., 2022). The resulting D7H-NPs displayed a uniform size of 46 ± 2 nm and exhibited superior water solubility. Especially, D7H-NPs could specifically gather in the lysosomes of 4T1 tumor cells, suggesting their ability of lysosome-targeted bioimaging. Moreover, the extensive production of •O_2_^−^ and ^1^O_2_ derived from D7H-NPs was noticed upon light excitation, thereby triggering tumor cell apoptosis. This work contributes a promising phototherapeutic agent based on helicenes concerning type I/II PDT, manifesting a facile strategy for organelle-targeted enhanced PDT therapy.

On account of light-triggered lysosomal disruption is beneficial for tumor therapy, a photo-activable theranostic nanoplatform (TPIMBS NPs) based on a synthetic multifunctional molecule (TPIMBS, chemical structure containing a cyanine dye core, organosilver moiety, and PEGylated segment) was constructed for spatiotemporally-controllable synergistic tumor therapy (Figure 8C) (Liu X. et al., 2023). With rational design, the synthetic TPIMBS photosensitizer exhibited function of aggregation-induced NIR emission and utilized organosilver as a chemotherapeutic agent. The amphiphilic block copolymers (DSPE-PEG-2000) at a mass ratio of 1:3 (TPIMBS:polymer) was introduced to assisted TPIMBS self-assemble into nanoparticles, rendering TPIMBS NPs a prolonged blood circulation half-life time of 12.5 h as well as expanded tumor accumulation *via* passive targeting. Through photochemical internalization, TPIMBS NPs favorably focused on the lysosomes of tumor cells and created ROS under light irradiation, leading to lysosomal disruption and release of Ag^+^ and cathepsin B from TPIMBS NPs. Cathepsin B contributes to both mitochondrial damage and inflammasome activation *via* the NLRP3 inflammasome/caspase-1/GSDMD-N pathway. Moreover, the release Ag^+^ could serve as a chemotherapeutic agent, which inhibits thioredoxin reductase and DNA polymerase to block multiple enzymatic pathways and result in cell apoptosis, significantly boosting the systemic antitumor efficacy by synergism with PDT (tumor inhibition rate = 91% vs. 65% for monotherapy). Notably, TPIMBS NPs with excellent stability can persevere accumulation at the tumor site for up to 36 h for prolonged bioimaging, and possess the desirable capability for tumor elimination in *in vivo* experiments. This nanoplatform exploits the combination of lysosomal-targeted ROS production with the photoactivable release of chemotherapeutic Ag^+^, to realize a spatiotemporally-controllable antitumor effect.

Accumulating evidence demonstrates that regular lysosomal function plays a critical role in autophagic degradation and the activation of pyroptosis (Liu Y. et al., 2024; Zhu et al., 2025). Inspired by the function of lysosome closely related to pyroptosis and autophagy, Sun *et al.* rationally synthesized a boron-dipyrromethene dimer (BDPd) comprising two lysosomal-targeted morpholine groups as NIR photosensitizer to augment PDT/PTT (Figure 8D) (Sun et al., 2024). To facilitate internalization into cancer cells, the synthesized BDPd was further self-assembled together with amphiphilic triblock copolymer (Pluronic F127) to prepare nanosized micelles (named BDPd NPs). BDPd NPs exhibited good biocompatibility, high ROS production yield, and excellent photothermal abilities. The perfect lysosomal-targeting capability endows BDPd NPs accumulating in lysosomes and stimulates vigorous lysosomal damage by boosted ROS under NIR laser irradiation, thus increasing the killing efficiency of 4T1 cancer cells. Mechanistically, BDPd NPs could efficiently damage the lysosomal and mitochondrial of cancer cells, activate ICD marker exposure, and trigger pyroptosis through simultaneously stimulating caspase-3/GSDME and NLRP3/GSDMD pathway, subsequently promoting DCs maturation. More importantly, the destruction of lysosomal functions in-turn blocks self-protective autophagic degradation, thus losing cytoprotection towards cancer cells. Either intratumoral or intravenous injection, BDPd NPs substantially suppressed tumor growth upon NIR light activation, provoked robust antitumor immune responses, and prevented tumor recurrence in breast tumor-bearing mouse models. Collectively, this work highlights the close relationship between subcellular organelles and PTT/PDT with advanced nanotechnology, to amplify the therapeutic outcomes in the future.

## 5 Targeting nucleus for enhanced PDT

Metabolism imbalance advances the tumor aggressiveness and resistance towards therapies, therefore cell nucleus regulating cellular metabolism and heredity becomes a brightening subcellular therapeutic location (Pham et al., 2023). Nucleus-targeted photosensitizers can apply pressure to DNA replication, facilitate apoptosis of cancer cells, as well as provoke inflammatory cells *via* secreting extensively proinflammatory factors and stimulate antitumor immune response (Liu et al., 2021). Targeting the DNA damage response and DNA repair capacity in cancer cells has been recognized as an important anti-cancer strategy in recent years (Chen M. et al., 2023). Accordingly, manners to target the nucleus of tumor cells are considered a perfect target for opening the complete potential of tumor phototherapy (Wang P. et al., 2024). However, nucleus-targeting drugs with high efficiency and biosafety characteristic are recently rare.

**FIGURE 9:**
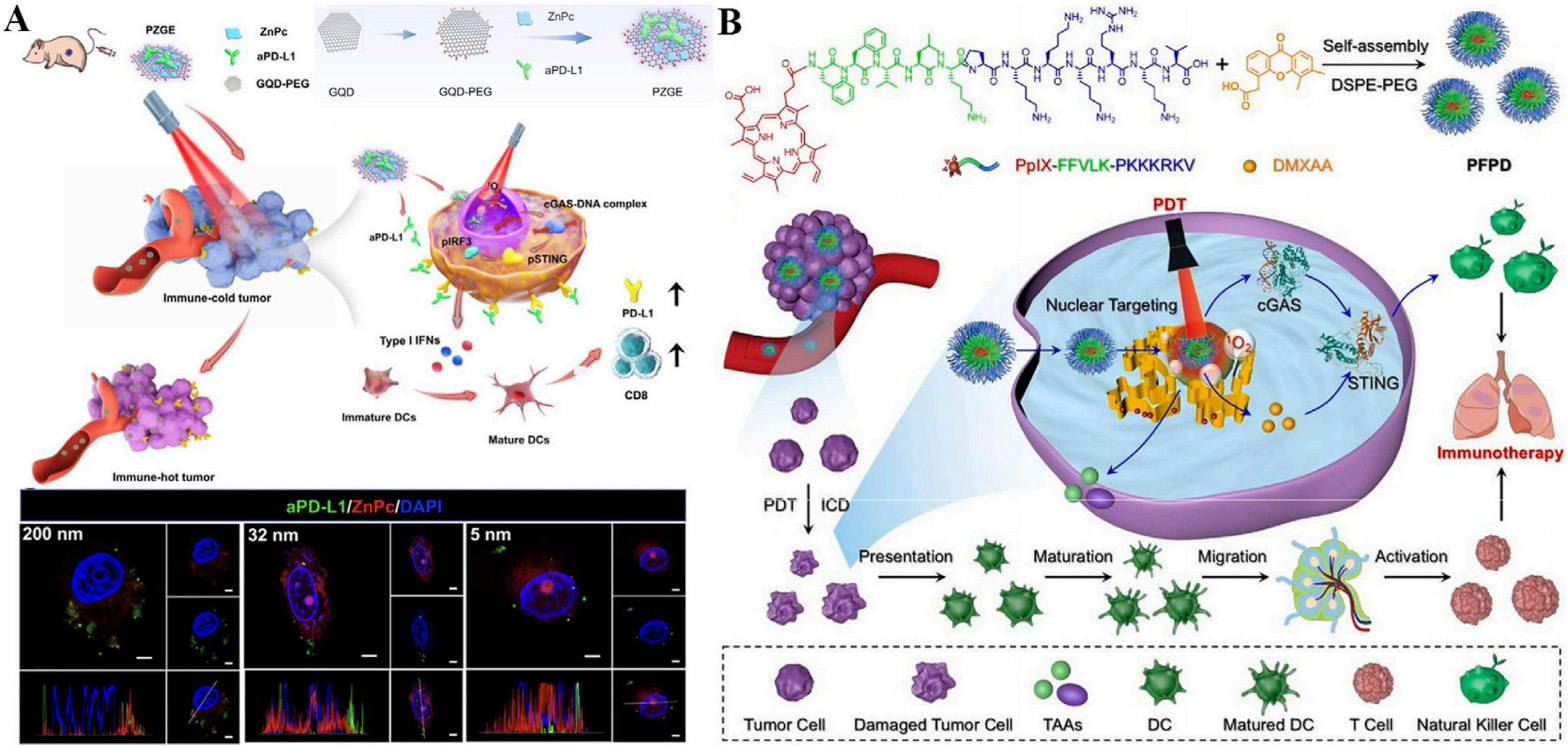
**(A)** Schematic illustration of PZGE for nucleus-targeted PDT-mediated photoimmunotherapy. Reproduced with permission from Ref. Zhang et al. (2023c). Copyright ^©^ 2023 Elsevier Ltd. **(B)** Schematic diagram of PFPD with nucleus-targeting ability for improved innate antitumor immunity *via* nucleus-targeted PDT and STING stimulation. Reproduced with permission from Ref. Wu et al. (2024b). Copyright ^©^ 2024 Elsevier Ltd.

Encouraged by nucleus-targeting photosensitizers-mediated PDT can elicit innate immune response for long-term immunotherapy, the combination of aPD-L1 with photoimmunotherapy is promising for reversing the immunosuppressive microenvironment and minimizing tumor recurrence (Zhang et al., 2023c). Zhang *et al.* constructed a novel nanomaterial named as PZGE by employing graphene quantum dots (GQDs) to loaded PD-L1 molecular antibody (aPD-L1) and zinc phthalocyanine (ZnPc) to promote innate immune response and transpose immunosuppressive microenvironment (Figure 9A) (Zhang et al., 2023c). To investigate the relationship between nucleus entry capacity and nanoparticle size, PZGE with three different particle sizes (5 nm, 32 nm, 200 nm) were synthesized. Then, the CCK-8 assay was employed to quantify their phototoxicity (Xiong et al., 2020; Cheng et al., 2021; Hu et al., 2021). Notably, PZGE with particle size of 5 nm exhibited the best nucleus enrichment ability due to its ultrasmall size, and amplified DNA destruction by nucleus-located PDT. Antitumor immunity was activated v*ia* cGAS/STING/IFN I pathway, followed by cytotoxic T lymphocyte infiltration and PD-L1 expression reversion. Besides, PZGE upon light irradiation effective inhibited the oral squamous cell carcinoma *in vivo*. Immunotherapy has introduced innovative design concepts and broad application prospects for the treatment of malignant tumors (Ge et al., 2023; Huang et al., 2023). The combination of phototherapy and immunotherapy offers a promising new approach in the fight against cancer (Liang et al., 2023). This work displays a size-dependent strategy of nucleus-targeted PDT and provides new insight into nucleus-targeting photo-mediated immunotherapy for “immune cold” tumors.

The combination of PDT and STING agonists has been shown to strongly augment the efficacy of antitumor therapy, so local delivery of photosensitizers and STING agonists based on nanoplatforms is expected to maximize tumor elimination (Ding et al., 2024). A nucleus-targeted chimeric peptide nanorod referred PFPD was constructed for antitumor precision therapy, which could enhance innate immunity through PDT-mediated local DNA damage and STING activation. Utilizing intermolecular forces, a photosensitizer (protoporphyrin IX, PpIX) was coupled onto multifunctional FFVLKPKKKRKV peptide and a novel chimeric peptide (PpIX-FFVLK-PKKKPRV) produced, which was self-assembled to form nanorods and loaded with DMXAA (a STING agonist) (Figure 9B) (Wu Y. et al., 2024). PFPD has a uniform and small size distribution with good stability, rendering its intratumor accumulation, cell internalization, and nucleus-targeted delivery of payloads. When exposure to laser, large amounts of ^1^O_2_ produced by PFPD induces *in situ* DNA fragmentation in nucleus, while the released cytoplasmic DNA fragments subsequently combined with the STING agonist to stimulate innate antitumor immunity. In addition, PFPD triggered ICD effect through nucleus-localized PDT, activated natural killer cells (NKs) and T cells, thus effectively eradicating primary and metastatic tumors with ignorable side effects. This research offers valuables for precise drug delivery and combination therapy against metastatic tumors with poor prognosis.

To deliver drugs to the nucleus of tumor cells more accurately, a hierarchical targeting cascade system towards tumor cells and the nucleus has been developed for tumor eradication through self-enhancement of PDT and chemotherapy. Specifically, a nucleus-targeting nanoprodrugs named HPC-CAT/CL were prepared based on human serum albumin (HSA), Ce6, a cisplatin prodrug (Pt (IV)), catalase (CAT), and b-cyclodextrin-lysine (CL) (Yang et al., 2023c). Then, the surface functionalization of HPC-CAT/CL was performed by a targeting agent (AS1411 aptamer), and the resulting HPC-CAT/CL-AP could actively target tumors, thus achieving self-reinforcing cascade photochemotherapy. HPC-CAT/CL-AP were efficiently taken up by tumor cells and accumulated in the nucleus, and non-toxic Pt (IV) prodrug was effectively reduced to its active state Pt (II) and supplied H_2_O_2_ simultaneously when exposed to overexpressed GSH in tumor cells and nucleus. Noteworthy, H_2_O_2_ could be decomposed by CAT to produce O_2_, which effectively alleviated the influence of anaerobic environment on the curative effect of PDT, and accordingly realized the synergistic photochemotherapy effect. The *in vitro* and *in vivo* experiments showed that HPC-CAT/CL-AP was highly successful to kill tumor cells, significantly repressing tumor growth and lung metastasis. This nucleus-targeted self-augmenting cascade photochemotherapy strategy has enormous potential in overcoming recurrence and metastasis of tumors.

Beyond traditional nucleus-targeting strategies (*e.g.*, size control, peptide-mediated delivery, and oligonucleotide aptamers targeting nuclear proteins), recent advances in s in nucleus-targeted PDT have focused on specific recognition of nuclear nucleic acids, including G-quadruplexes (G4s) and general RNA/DNA, to enhance targeting precision and therapeutic efficacy (Long et al., 2024; Chen W. et al., 2025).​ G4s are stable secondary structures formed by guanine-rich nucleic acids in oncogene promoters and telomeres, making them attractive targets for selective tumor intervention (Liu et al., 2024a). Building on this foundation, an advanced donor-π-acceptor (D-π-A) acridinium-based Type I photosensitizer was developed for RNA G4s targeting (Chen W. et al., 2023). Its structure integrates triphenylamine (electron donor) and pyridinium (electron acceptor) to achieve NIR absorption and efficient electron transfer. This acridinium derivative specifically recognizes RNA G4s through hydrogen bonding, with negligible affinity for double-stranded DNA or single-stranded RNA. Upon light irradiation, it predominantly generates •O_2_^−^, instead of ^1^O_2_, overcoming hypoxia-induced PDT resistance and enhancing ICD in tumors.

Additionally, direct targeting of nuclear RNA/DNA *via* sequence complementarity or intercalation has also advanced PDT precision. A nucleus-targeted activatable photosensitive probe (CMT-I) was designed and synthesized for DNA targeting (Liao et al., 2025). Molecular docking analyses indicate that CMT-I binds to DNA selectively, relying on hydrogen bonds and π-π conjugation for interaction. *In vitro* spectral tests show that ct-DNA specifically activates CMT-I, leading to a notable fluorescence enhancement upon binding. When irradiated with light, CMT-I proves effective at generating ^1^O_2_ RNA sequencing studies reveal that the PDT induced by CMT-I activates tumor cell immunity, initiating an adaptive immune response. *In vivo* therapeutic trials further confirm that CMT-I boosts antitumor immunity, a key factor in successfully eliminating immunologically cold tumors, and underscores the value of nucleus-targeted PDT for precise cancer treatment. Together, these G4-and nucleic acid-targeted approaches represent a paradigm shift from broad nuclear localization to molecularly precise damage, with successive advancements significantly improving PDT efficacy and reducing systemic toxicity.​

## 6 Targeting cell membrane for enhanced PDT

Due to ROS possess a short half-life and a restricted effective diffusion radius, the introduction of photosensitizers to produce ROS *in situ* on target organelles is essential to avoid their rapid decay. Among these, the plentiful unsaturated lipids of the plasma membrane can stimulate LPO under the action of ROS produced by PDT, thus damaging the cell integrity (Fan et al., 2021). Therefore, photosensitizer-based cell membrane-targeted delivery systems can improve membrane permeability, cause LPO, disrupt membrane integrity, and ultimately result in cell membrane disruption and release of cell payloads (Wang M. et al., 2022). In recent years, membrane-targeted photosensitizer systems have been established, which could deliver photosensitizers to therapeutic target organelles to enhance the efficiency of PDT (Yang et al., 2023b).

**FIGURE 10:**
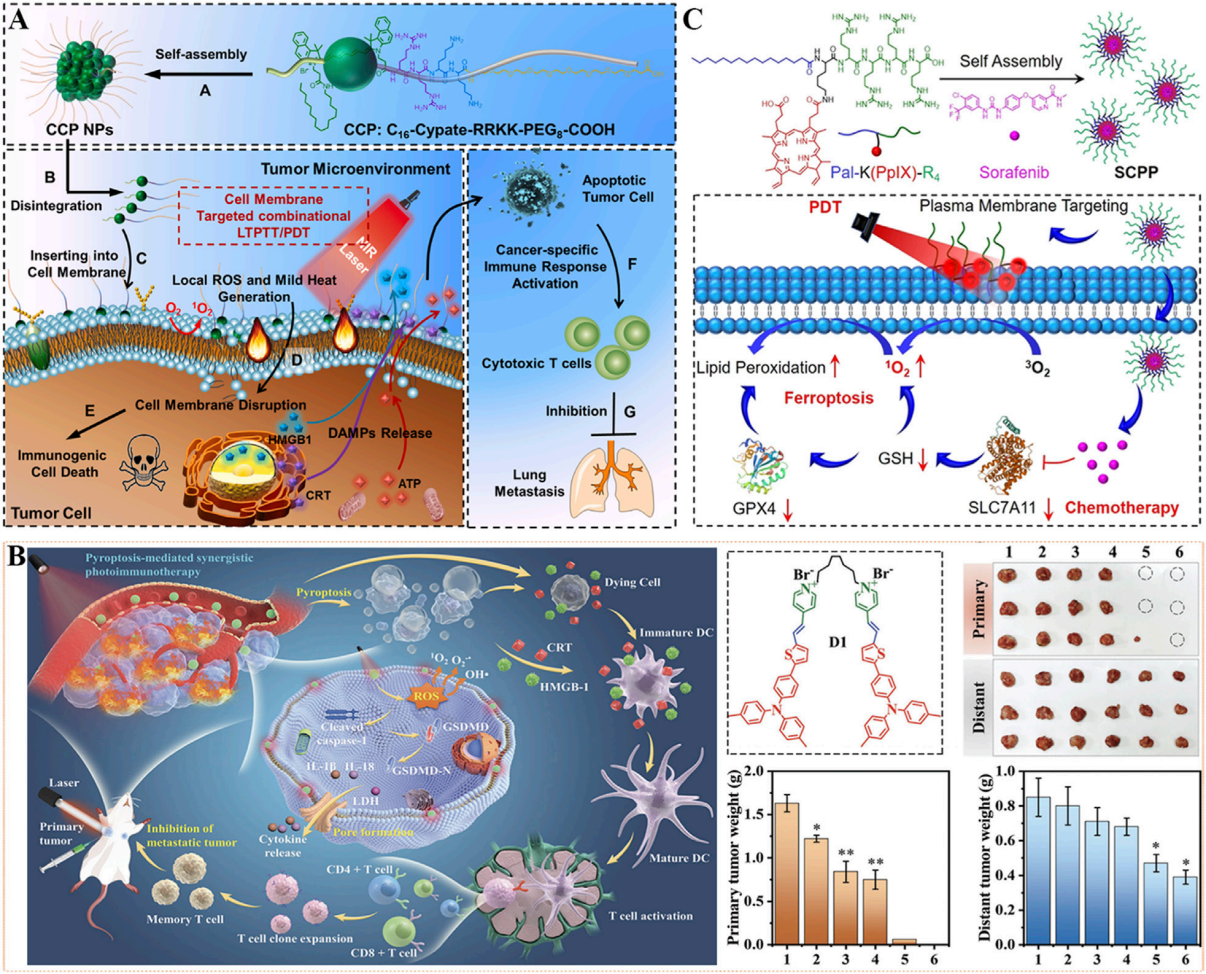
**(A)** The illustration of cell membrane-targeting CCP NPs for antimetastatic combination low-temperature PTT/PDT therapy. Reproduced with permission from Ref. (Chen et al., 2022). Copyright © 2022 Elsevier Ltd. **(B)** Schematic illustration of photosensitive dimer D1-based pyroptosis-mediated synergistic photodynamic and photothermal immunotherapy for inhibition of primary and distant tumor. 1: Control, 2: Laser, 3: M1, 4: D1, 5: M1+Laser, 6: D1+Laser. M1: monomer. Reproduced with permission from Ref. Tang et al. (2023). Copyright © 2023 WILEY-VCH Verlag GmbH & Co. KGaA. **(C)** The preparation and antitumor mechanism of SCPP utilized for plasma membrane-targeted combination therapy. Reproduced with permission from Ref. Deng et al. (2022). Copyright © 2022 American Chemical Society.

To amerliorate phototherapy-mediated tumor metastasis suppression, self-delivery chimeric peptide (C_16_-CypateRRKK-PEG_8_-COOH, CCP) was designed for cell membrane-targeted low-temperature PTT and PDT combination therapy. As depicted in Figure 10A, CCP was constructed from palmtic acid-modified NIR dye (Cypate, hydrophobic) and chimeric peptide Arg-Arg-Lys-Lys-PEG_8_ (RRKK-PEG_8_-COOH, hydrophilic), whose amphiphilicity gives it the ability to self-assemble into nanoparticles (CCP NPs) (Chen et al., 2022). Among them, Cypate is a photosensitive agent for clinical use with dual effects of PDT and PTT (Zou et al., 2025b), positively charged RRKK fragment and alkyl chain of palmitic acid enable CPPs to target and insert into the cell membrane, and PEG chain endows prolong the blood circulation time of CCP NPs. CCP NPs exhibited strong tumor targeting and accumulation capability, which could effectively kill tumor cells. Under NIR laser irradiation, CCP NPs can produce numerous ROS and mild heat of less than 45 °C locally in the cell membrane, destroy the cancer cell membrane, effectively induce ICD, and activate antitumor immune response, thereby eliminating residual tumor cells. Under single administration and NIR light irradiation, CCP NPs significantly inhibited tumor growth and lung metastasis, thus extending the survival time of mice. This unique cell membrane-targeted phototherapy strategy helps to achieve tumor clearance and inhibit metastasis in a safe and effective manner.

As a form of programmed cell death caused by inflammasome, pyroptosis is an important means to overcome drug resistance towards apoptosis and fight against immunosuppressive tumor microenvironment, and ultimately inhibit tumor growth (Loveless et al., 2021; Zhu Y. et al., 2024). However, the current pyroptosis-induced drugs have some limitations, such as high toxicity, poor stability, and low intracellular accumulation. In view of this, a cell membrane-targeting photosensitizer dimer (D1) with aggregation-induced emission properties was constructed for synergistic pyroptosis-mediated photodynamic and photothermal immunotherapy. In detail, a *π*-conjugated chromophore with strong intramolecular charge transfer ability was formed by thiophene, triphenylamine, and pyridinium groups, and then linked two chromophores with one flexible octyl group to construct the dimeric photosensitizer D1 (Figure 10B) (Tang et al., 2023). This photosensitive dimer D1 could specially accumulate on the cell membrane, and generate ROS by type I PDT as well as heat energy by PTT under light irradiation, enabling a reinforced pyroptosis effect. Additionally, the pyroptosis triggered by synergistic phototherapy effectively triggered the discharge of inflammatory cytokines and intracellular contents, promoted tumor-specific antigen induction and DCs maturation, and stimulated T cell proliferation, thus activating systemic antitumor immunity. In mechanism, D1 exhibits improved permeability and retention effect in the tumor cell membrane, high ROS-generated efficiency, and strong photothermal effect, which accelerates pyroptosis-mediated synergistic PDT/PTT/immunotherapy in mouse models. After 7 days of treatment with laser irradiation, D1 was found to completely eliminate the primary tumors and effectively inhibit the distant tumors. This work provides a promising strategy for a satisfactory antitumor effect and rouses systemic immune response against tumor metastasis.

By self-assembly of chemotherapeutic agent sorafenib and plasma membrane-targeted amphiphilic chimeric peptide (Pal-K(PpIX)-R_4_), Deng *et al.* have successfully prepared a nanoscale photooxidant named as SCPP (Figure 10C) (Deng et al., 2022). Among them, the palmitic part and positively charged peptide of Pal-K(PpIX)-R_4_ (hydrophobic) given the SCPP plasma membrane-targeting capability. SCPP displayed excellent stability and ideal size distribution, which could enhance localized LPO production by *in situ* PDT with laser. In addition, intracellular accumulation of the chemotherapy drug (sorafenib) can downregulate the levels of GSH and glutathione peroxidase 4 (GPX4) by blocking the expression of cystine/glutamate antiporter (SLC7A11), thereby amplifying the PDT effect and disrupting the antioxidant defense system. Therefore, SCPP could trigger LPO through plasma membrane-targeting PDT, and activate ferroptosis of tumor cells through sorafenib-mediated chemotherapy, finally exhibiting superior tumor inhibition ability *in vivo*. This work may afford scientific direction for plasma membrane-targeted synergistic treatment of tumors when exposure to disadvantage conditions.

## 7 Targeting ribosome for enhanced PDT

**FIGURE 11:**
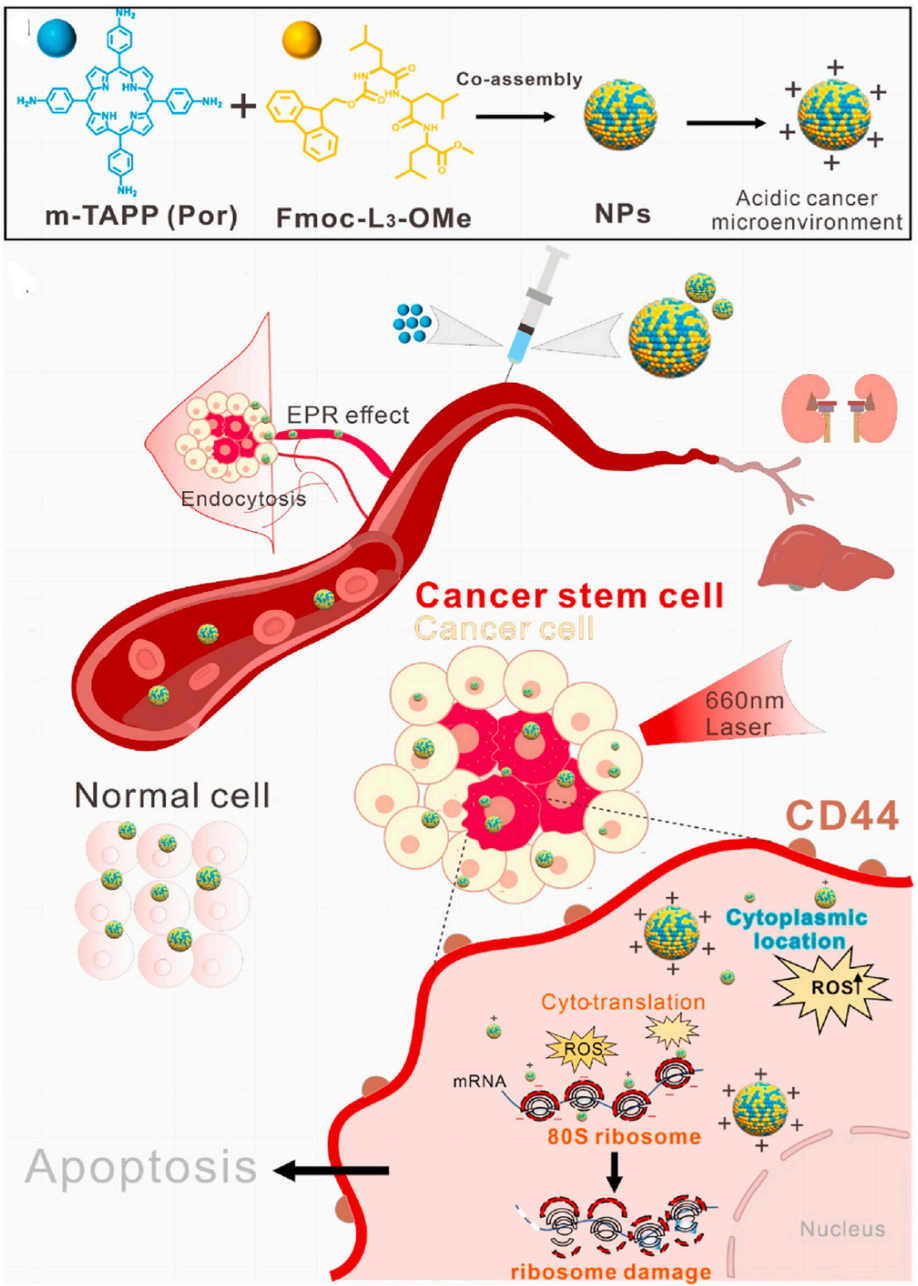
Schematic illustration that showing NPs targeted to cytosolic ribosomes and inhibited the CSCs upon light irradiation. Reproduced with permission from Ref. Wang et al. (2022a). Copyright ^©^ 2022 Elsevier Ltd.

Ribosomes are highly complex cellular machines, primarily composed of ribosomal RNA (rRNA) and dozens of different ribosomal proteins (R-proteins); the exact number varies slightly between species (Cheng et al., 2024). These ribosomal proteins and rRNA are arranged into two subunits of different sizes, commonly referred to as the small and large subunits of the ribosome (Elhamamsy et al., 2022). These subunits work together to convert mRNA into polypeptide chains during protein synthesis (Jiao et al., 2023). It has been reported that cancer stem cells (CSCs) have a higher abundance of ribosomes than those found in normal cells, which is one of the important markers of CSCs (Xue et al., 2024). The proportion of ribosomes in the body determines the total proteome and the rate of cell division, thus influencing the cell cycle and its fate. Due to the negative charges of ribosomes, positively charged materials interact strongly with ribosomes to achieve targeting. Qin’s group (Figure 11) first synthesized a positively charged photosensitizer (meso-Tetra (4-aminophenyl)Porphyrin, m-TAPP) and small peptides (N-fluorenylmethoxycarbonyl-leucine-leucine-leucine-OMe, Fmoc-L3-OMe) that co-assemble into nanoparticles based on π-π interactions (Wang J. et al., 2022). The pH of tumor cells is 6.5 lower than that of normal cells, which allows the positively charged co-assembly to interact strongly with ribosomes for effective targeting. The co-assembly exhibits strong ROS production under light irradiation, significantly reducing tumor stem cells and deactivating ribosomes, and inhibiting tumor cell growth both *in vitro* and *in vivo*.

## 8 Targeting endoplasmic reticulum for enhanced PDT

The ER is a multifunctional organelle responsible for maintaining cell homeostasis, protein synthesis, folding, and secretion. The production of ROS by PDT could disrupt the protein-folding capacity of this organelle and trigger ER stress, which leads to tumor cell death (Diao et al., 2025). Therefore, targeting the ER is critical to evoke ER stress.

### 8.1 Paraxin

**FIGURE 12:**
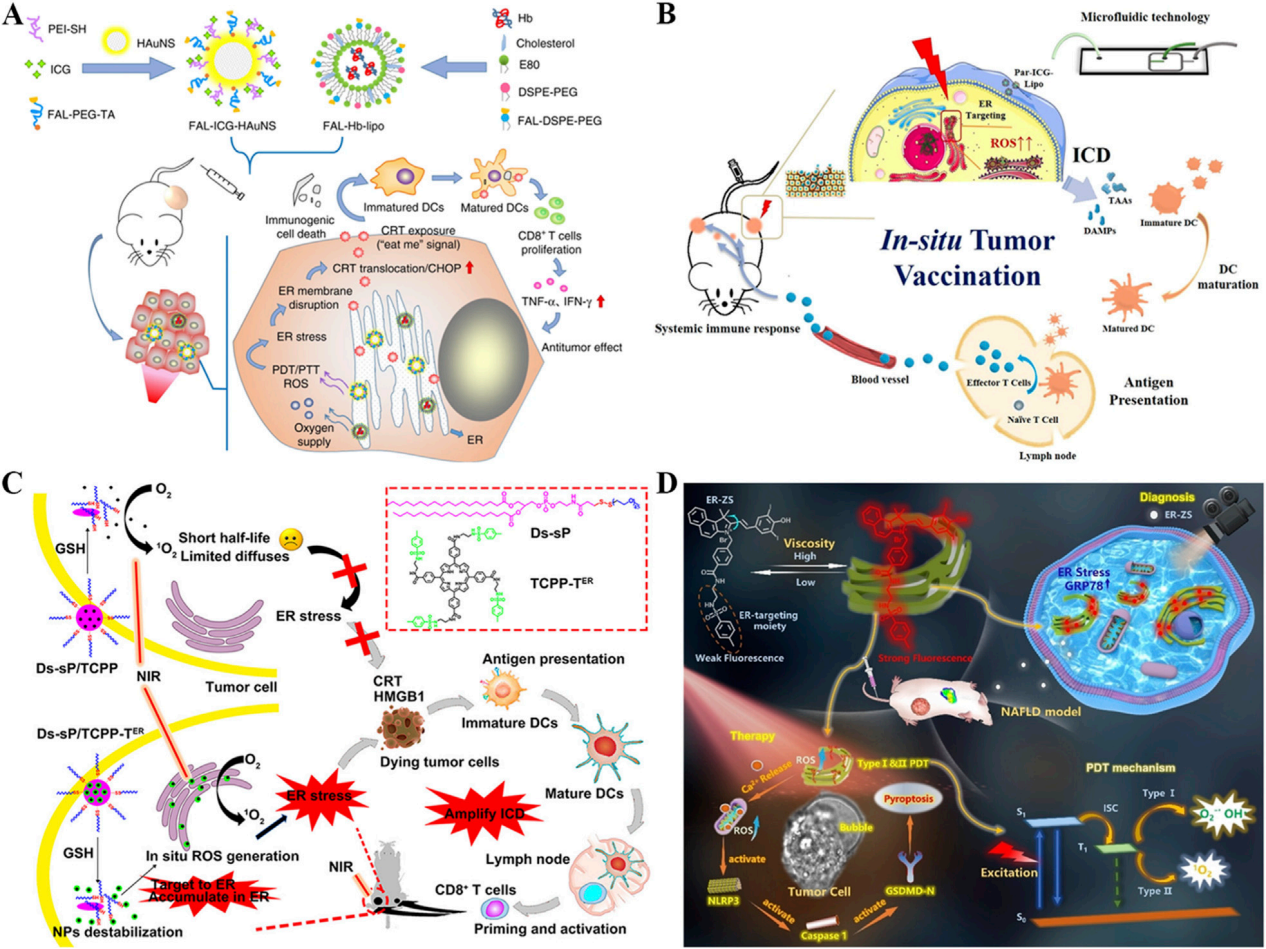
**(A)** Schematic illustration of enhanced immunogenic cancer cell death and anticancer effect induced by ER-targeting photothermal/photodynamic therapy. Reproduced with permission from Ref. Li et al. (2019). Copyright ^©^ 2019 Springer Nature. **(B)** Graphic illustration of Pardaxin-ICG-Liposome mediated ER-targeting PDT under NIR irradiation, which could induce immunogenic tumor cell death, acting as an *in situ* tumor vaccination. Reproduced with permission from Ref. Liu et al. (2022b). Copyright ^©^ 2022 American Chemical Society. **(C)** Schematic diagram of efficient ER-targeting PS TCPP-TER to induce ER stress and amplify ICD. Reproduced with permission from Ref. Deng et al. (2020a). Copyright ^©^ 2020 American Chemical Society. **(D)** Schematic illustration depicting the molecular mechanisms of ER-ZS for diagnosing NAFLD, and for photodynamic cancer therapy by activating tumor cell pyroptosis. Reproduced with permission from Ref. Zeng et al. (2024b). Copyright ^©^ 2024 Wiley-VCH GmbH.

Pardaxin, served as ER-targeting peptides, combined with a photosensitizer, were delivered to the ER to induce ICD *via* ER stress. You *et al.* designed pardaxin-modified and indocyanine green (ICG) conjugated multifunctional nanoparticles (Figure 12A), as well as O_2_-carrying hemoglobin (Hb) liposomes (FAL-Hb lipo) to reverse hypoxia (Li et al., 2019). In the hypoxic tumor environment, this system showed a special affinity for the ER and produced ^1^O_2_ species under light irradiation. The ER-localized ^1^O_2_ species triggered strong ER stress and calreticulin (CRT) exposure on the cell surface as an “eat me” signal. CRT, a biomarker of ICD, promotes the maturation of dendritic cells (DCs) and further induces an immunological response, including CD8^+^ T cell proliferation and cytotoxic cytokine secretion. Finally, the ER-targeting nanosystem achieves enhanced antitumor effects. In 2022, this group reported another ER-targeting nanoparticle (Pardaxin-ICG-Liposome) (Figure 12B) fabricated through microfluidic techniques (Liu X. et al., 2022). Under near-infrared (NIR) irradiation, Pardaxin-ICG-Liposome could produce an ICD effect and activate the immune system by ER-targeting PDT.

### 8.2 p-Toluenesulfonamide

p-Toluenesulfonamide has been reported to target ER by binding to the sulfonamide receptor (Zhu et al., 2021). Song *et al.* synthesized reduction-responsive Ds-sP nanoparticles encapsulating an ER-targeting photosensitizer (Figure 12C), TCPP-TER (Deng H. et al., 2020). The Ds-sP/TCPP-TER nanoparticles could accumulate in the ER and generate ROS to trigger ER stress under 670 nm laser irradiation, resulting in an enhanced ICD effect. The amplified ICD then induced dendritic cell (DC) activation, leading to an augmented immune response, including the infiltration of CD8^+^ T cells and enhanced secretion of cytokines. Therefore, the ER-targeting Ds-sP/TCPP-TER exhibited a better antitumor effect compared to Ds-sP/TCPP. Additionally, Yoon *et al.* designed p-Toluenesulfonamide-modified hemicyanine dyes (ER-ZS) (Figure 12D), which are type I photosensitizers, capable of producing •O_2_^−^ and •OH radicals under light irradiation (Zeng et al., 2024b). The numerous ROS generated in the ER could effectively damage it, activating the pyroptosis cascade pathway, and subsequently causing DNA breakage and effectively inhibiting tumor growth.

### 8.3 Others

**FIGURE 13:**
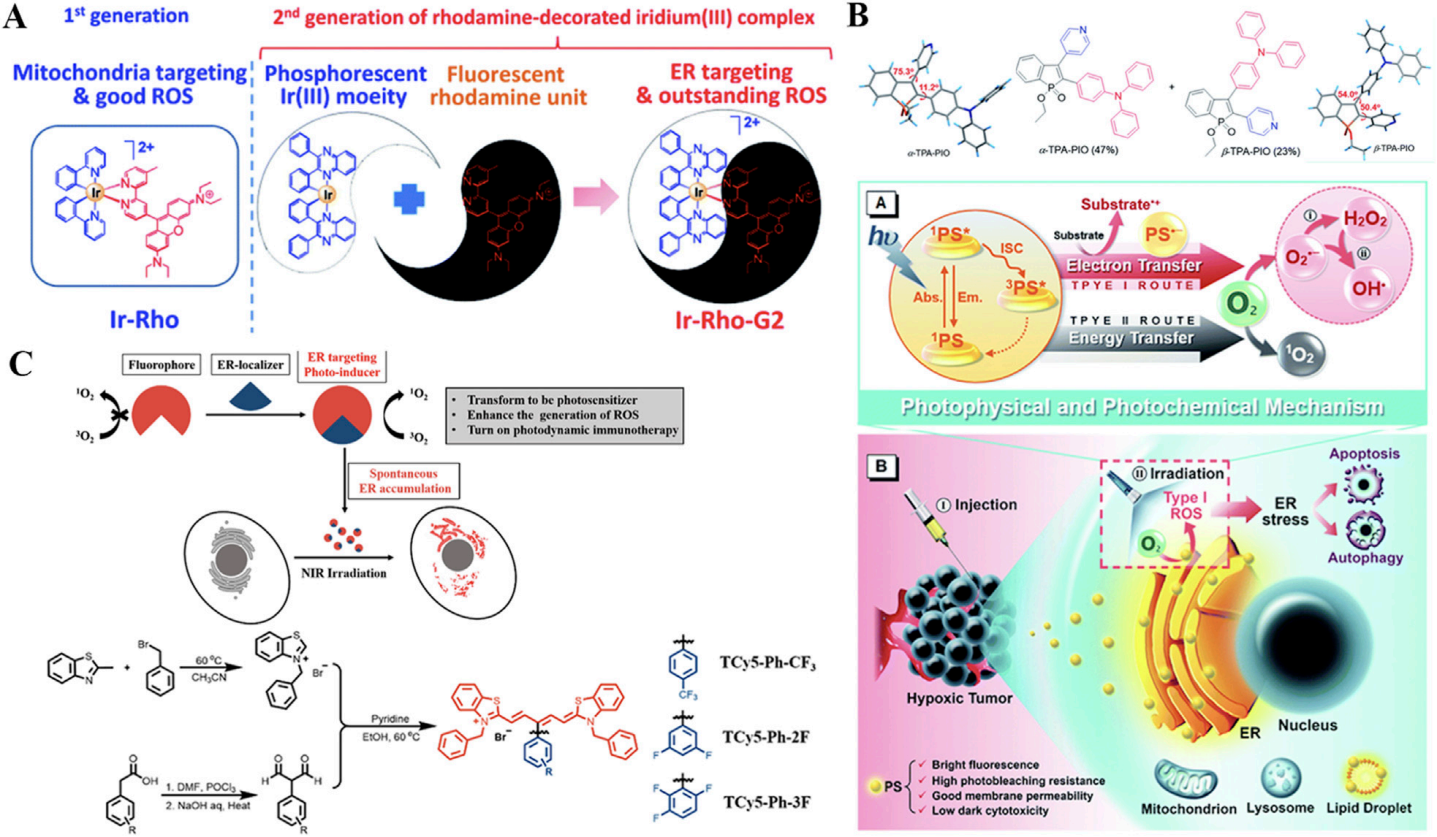
**(A)** Schematic illustration of rhodamine-decorated iridium (III) complex, Ir-Rho-G2, with ER targeting and enhanced ^1^O_2_ generation showing high efficiency-induced apoptosis of cancer cells for efficient PDT. Reproduced with permission from Ref. Zhou et al. (2020). Copyright ^©^ 2020 The Royal Society of Chemistry. **(B)** Schematic illustration of the cytological process of PDT treatment mediated by PIO-based fluorogens, and the structures of α-TPA-PIO and β-TPA-PIO. Reproduced with permission from Ref. Zhuang et al. (2020). Copyright ^©^ 2020 The Royal Society of Chemistry. **(C)** Schematic of the ER localizer mediating precise construction of the photosensitizer for the ER and the synthesis of TCy5 derivatives. Reproduced with permission from Ref. Ma et al. (2022). Copyright ^©^ 2022 American Chemical Society.

Wong and colleagues developed a rhodamine-decorated iridium (III) complex by modifying the cyclometallating ligand to enhance the production of ROS and to achieve specific localization in the ER (Figure 13A) (Zhou et al., 2020). The replacement of the bipyridine (ppy) chelating ligand with 2,3-diphenylquinoxaline (dpqx) resulted in the complex Ir-Rho-G2, which demonstrated a higher ^1^O_2_ generation quantum yield and a lower luminescence quantum yield compared to Ir-Rho, thereby enhancing its ROS generation capability. Additionally, the dpqx ligand in Ir-Rho-G2 conferred a predominant and specific intracellular localization to the ER. Consequently, the cytotoxicity of Ir-Rho-G2 was approximately three times greater than that of Ir-Rho under lamp irradiation, attributable to its ability to disrupt protein folding in the ER and to activate ER stress-induced apoptosis pathways. *In vivo* PDT experiments, Ir-Rho-G2 effectively targeted the tumor site and suppressed tumor growth.

Tang’s group (Figure 13B) described two Type I photosensitizers, α-TPA-PIO and β-TPA-PIO, both derived from phosphindole oxide (Zhuang et al., 2020). These photosensitizers demonstrated selective accumulation in the ER, with β-TPA-PIO exhibiting a higher tumor inhibition efficiency under light irradiation than α-TPA-PIO, owing to its enhanced ROS generation capacity. *In vivo*, β-TPA-PIO was shown to effectively destroy tumor cells while maintaining good biocompatibility. The ER stress elicited by the excessive production of local ROS could activate apoptosis and autophagy pathways, thereby contributing to a significant PDT effect.

Peng *et al.* designed three types of fluorinated thio-pentamethine dyes, with TCy5-Ph-3F exhibiting the highest ^1^O_2_ generation efficiency and cell killing ability compared to TCy5-Ph-2F and TCy5-Ph-CF3 (Figure 13C) (Ma et al., 2022). The half-maximal inhibitory concentration (IC_50_) of TCy5-Ph-3F was 125 nM following LED light irradiation for 10 min, significantly lower than that of TCy5-Ph-2F (IC_50_ ∼0.5 μM) and TCy5-Ph-CF3 (IC_50_ > 1 μM). TCy5-Ph-3F localized spontaneously to the ER with a Pearson correlation coefficient of 0.96. *In vitro* studies demonstrated that ROS production by TCy5-Ph-3F within the ER can induce enhanced ER stress and trigger the ICD cascade in cancer cells. *In vivo*, the efficacy of TCy5-Ph-3F in inhibiting primary and distant tumor growth under light irradiation exceeded that of the TCy5-Ph-2F group, attributable to an ER-targeting activated immune response.

## 9 Targeting golgi apparatus for enhanced PDT

The Golgi apparatus (GA) functions as a vital organelle in the intracellular membrane transport network, acting as a crossroads for extracellular and intracellular pathways (Xu et al., 2024). It is fundamental in the processing, sorting, and trafficking of proteins synthesized within the cell, ensuring their proper delivery to the intended destinations. The generation of ROS can adversely affect the structure and function of the GA, potentially leading to modifications in stress signaling pathways downstream (Hu et al., 2023). Such alterations may play a role in inhibiting tumor cell growth, contributing to the activation of cellular stress responses such as apoptosis or cell cycle arrest.

### 9.1 Chondroitin sulfate

**FIGURE 14:**
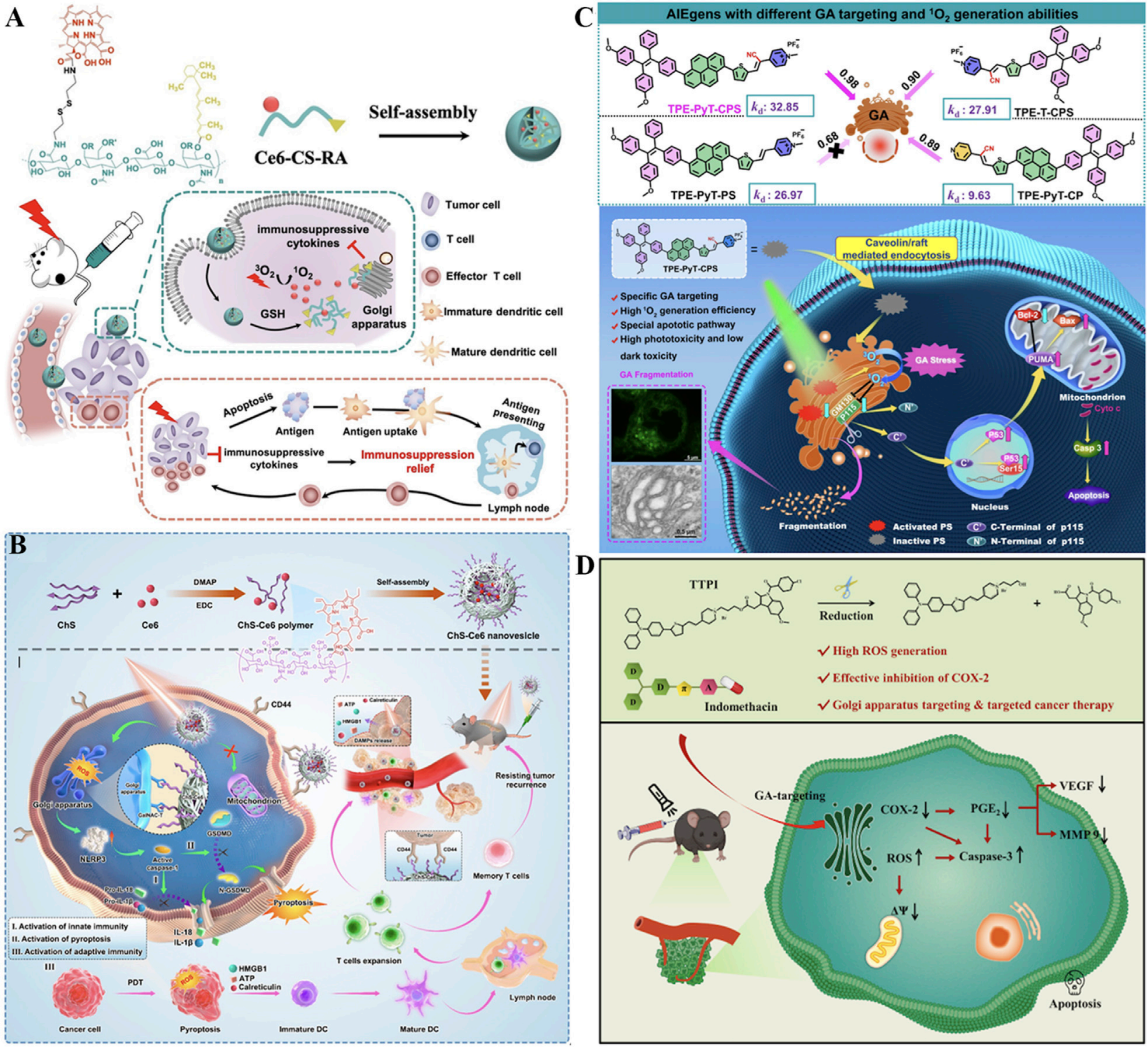
**(A)** Schematic illustration of the *in vivo* application of the Golgi apparatus-targeted prodrug Ce6-CS-RA nanoparticles for enhanced photodynamic immunotherapy. Reproduced with permission from Ref. Li et al. (2022a). Copyright ^©^ 2022 Elsevier Ltd. **(B)** A schematic representation of the preparation and use of Golgi apparatus-targeted ChS-Ce6 nanovesicles for the treatment of NSCLC-SM. Reproduced with permission from Ref. Hu et al. (2023). Copyright ^©^ 2023 American Chemical Society. **(C)** GA targeting and ROS generation capacity of the AIEgens, as well as the apoptosis pathway caused by Golgi oxidative stress. Reproduced with permission from Ref. Liu et al. (2022a). Copyright ^©^ 2022 Springer Nature. **(D)** COX-2 inhibition mediated TTPI exerted enhanced PDT to kill cancer cells. Reproduced with permission from Ref. Wang et al. (2024d). Copyright ^©^ 2023 Elsevier Ltd.

Chondroitin sulfate, a polysaccharide abundant in the extracellular matrix and on cell surfaces, possesses a sugar chain structure consisting of glucuronic acid and N-acetylgalactosamine (GalNAc) (Li H. et al., 2022). This structure enables it to bind to the surface receptor GalNAc-T1, specifically targeting the Golgi apparatus of tumor cells. For example, Gong and colleagues designed a nanodrug based on chondroitin sulfate that encapsulates the photosensitizer chlorin e6 (Ce6), retinoic acid (RA), and a CpG oligodeoxynucleotide (Figure 14A) (Li H. et al., 2022). The production of ROS by this nanodrug can damage tumor cells, and the immunosuppressive effects of PDT are mitigated by targeting and destroying the Golgi apparatus. In a subsequent study, Dong’s group developed chondroitin sulfate-based nanoparticles (ChS-Ce6) where the photosensitizer Ce6 was covalently attached to chondroitin sulfate (Figure 14B) (Hu et al., 2023). The ChS-Ce6 nanoparticles were directed to the Golgi apparatus due to the strong interaction between chondroitin sulfate and GalNAc-T1 on the Golgi apparatus. Consequently, tumor cell growth was significantly inhibited in the ChS-Ce6 group upon laser irradiation, as chondroitin sulfate enhanced ROS production by Ce6. This increase in ROS upregulated the nucleotide-binding oligomerization domain-like receptor family pyrin domain-containing 3 (NLRP3), promoting the release of inflammatory contents, generating an inflammatory environment, and triggering downstream classical caspase-1-dependent pyroptosis.

### 9.2 Others

While other materials for targeting the Golgi apparatus do exist, Guo’s group designed aggregation-induced emission (AIE) photosensitizers, termed TPE-PyT-CPS, specifically for targeting the Golgi apparatus (Figure 14C) (Liu M. et al., 2022). These photosensitizers, modified with a CN group, are capable of forming large rod-like aggregates ranging from 200 to 400 nm in size and can target the Golgi apparatus via caveolin/raft-mediated endocytosis. The TPE-PyT-CPS, with its Golgi apparatus-targeting capability, induces oxidative stress within the Golgi apparatus and triggers mitochondrial-dependent apoptosis. This study demonstrates that photosensitizers with the ability to target the Golgi apparatus can more effectively inhibit tumor growth, providing a novel strategy for precise and efficient tumor therapy.

**FIGURE 15:**
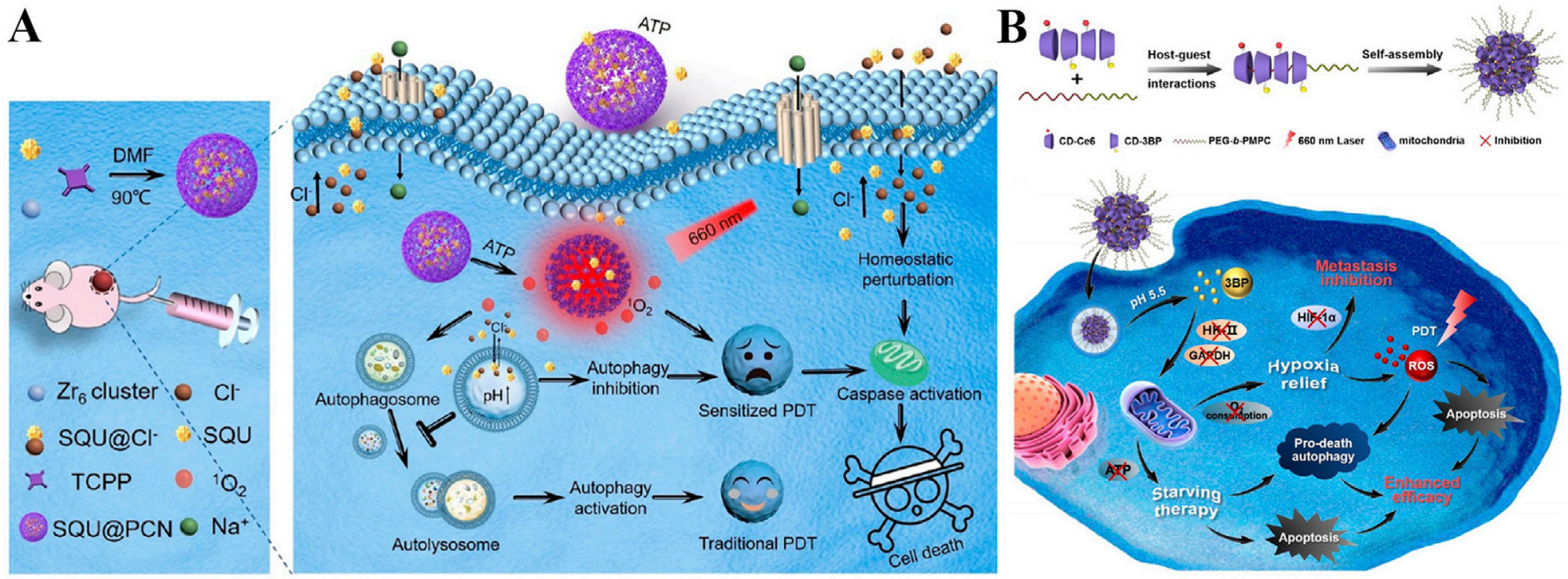
**(A)** Schematic Illustration of SQU@PCN Preparation and the Tumor Cell Death Process by Homeostatic Perturbation Therapy and Sensitized Photodynamic Therapy. Reproduced with permission from Ref. Wan et al. (2019). Copyright ^©^ 2019 American Chemical Society. **(B)** Schematics of the synthesis and fabrication of CD-Ce6-3BP NPs induced pro-death autophagy to enhance photodynamic therapy against hypoxic tumor. Reproduced with permission from Ref. Deng et al. (2020b). Copyright ^©^ 2020 American Chemical Society.

Qi’s group developed an aggregation-induced emission (AIE) photosensitizer, TPPI, which combines a cationic triphenylamine-based photosensitizer with the cyclooxygenase (COX-2) inhibitor Indomethacin (IMC) (Figure 15D) (Wang X. et al., 2024). COX-2 is overproduced and accumulated in the Golgi apparatus, allowing TPPI to specifically target the Golgi apparatus by recognizing COX-2 through IMC. Upon light irradiation, TPPI produces an abundance of ROS, leading to the destruction of the Golgi apparatus and cell death, thereby enhancing PDT through targeted action on the Golgi apparatus. Furthermore, Wu *et al.* created a Golgi apparatus-targeting Ru-SL complex consisting of Ru(II) pyridine and sphingosine lipid (Zhao et al., 2021). This complex effectively localizes within the Golgi apparatus and, upon light exposure, induces damage to the apparatus, resulting in the killing of tumor cells.

## 10 Targeting autophagosome for enhanced PDT

Autophagy is a highly conserved lysosomal-mediated degradation process in eukaryotes. Autophagosomes, which are double-membrane vesicles formed during autophagy, encapsulate autophagy substrates and transport them to lysosomes for degradation (Liu et al., 2024b). This process is crucial for cell survival, growth, and homeostasis. Reports indicate that PDT efficacy is enhanced in tumor cells when autophagy is suppressed by inhibitors. Zhang *et al.* developed an adenosine-triphosphate (ATP)-regulated ion transport nanosystem, comprising a porphyrinic porous coordination network (PCN) and squaramide (SQU) (Figure 15A) (Wan et al., 2019). The release of SQU in response to high ATP levels inhibits autophagy by disrupting cellular ion homeostasis, sensitizing PDT, and improving its therapeutic efficacy. Conversely, Jin’s group studied acid-sensitive CD-Ce6-3BP nanoparticles loaded with the respiration inhibitor 3-bromopyruvate (3BP) and the photosensitizer Ce6, facilitated by host-guest interaction (Figure 15B) (Deng Y. et al., 2020). 3BP reduces intracellular O_2_ consumption, alleviating tumor hypoxia, and converting autophagy from a pro-survival to a pro-death process. This excessive activation of autophagy enhances the PDT effect.

## 11 Conclusion

In recent years, PDT has emerged as a minimally invasive, highly regulable, and immunogenic therapeutic strategy that holds great promise in the field of cancer treatment. Despite its growing popularity and notable achievements in preclinical studies, several critical challenges remain before PDT can be widely adopted in clinical practice-particularly in improving therapeutic efficiency, enhancing targeting specificity, and overcoming tumor microenvironmental limitations. This review systematically summarizes recent advances in subcellular organelle-targeted PDT strategies aimed at enhancing therapeutic outcomes. Particular emphasis is placed on nanoplatforms designed to target mitochondria, lysosomes, the nucleus, cell membrane, Golgi apparatus, and ribosomes, with detailed discussion of their targeting mechanisms and underlying pathways that contribute to enhanced PDT efficacy. These approaches not only improve the accumulation of photosensitizers at specific organelles but also modulate key intracellular signaling and oxidative stress responses, thereby potentiating antitumor effects.

Although numerous studies have demonstrated the therapeutic potential of PDT, several key issues still need to be addressed. First, in terms of drug delivery, many currently used photosensitizers suffer from poor water solubility and limited stability *in vivo*, often requiring complex delivery systems for effective administration. This increases formulation complexity and may compromise bioavailability. Secondly, most photosensitizers inherently lack targeting ability and must be conjugated-either covalently or non-covalently-to targeting ligands such as peptides, antibodies, or small molecules to achieve precise subcellular localization. Such multi-step functionalization processes further complicate preparation and may lead to batch-to-batch variability. Moreover, the hypoxic tumor microenvironment remains a major obstacle to efficient ROS generation during PDT. While various strategies-such as O_2_-carrying materials, catalytic H_2_O_2_ decomposition, or mitochondrial respiration inhibition and regulation-have shown promising results in cellular and animal models, their safety and efficacy in humans remain to be fully validated. Additionally, most conventional photosensitizers rely on visible light excitation, which limits tissue penetration and restricts their application to superficial tumors. Therefore, developing photosensitizing systems responsive to NIR or X-ray irradiation could significantly expand the clinical applicability of PDT. Finally, the high interstitial pressure and dense extracellular matrix within solid tumors pose significant barriers to effective drug delivery. As a result, the concentration of systemically administered nanomedicines reaching the tumor site is often insufficient. To address this challenge, biomimetic delivery systems-such as cell membrane-coated nanoparticles or exosome-based carriers-along with active targeting strategies may offer promising solutions to enhance tumor accumulation and therapeutic performance.

From the clinical translation perspective, subcellular organelle-targeted PDT faces multiple bottlenecks hindering its transition from lab to clinic. Insufficient pharmacokinetic data on nano-photosensitizers impedes precise control of dosage and frequency, raising clinical risks. The lack of long-term biosafety assessments is a major concern, as some nanomaterials may accumulate *in vivo*, causing chronic toxicity or immune reactions that require long-term monitoring. Additionally, standardizing light irradiation doses in multi-center trials is challenging, in which variations in equipment, duration, and intensity reduce result comparability, hampering unified clinical standards.​ Notably, clinical experience with second-generation photosensitizers such as ALA in treating condyloma acuminatum, encompassing administration protocols, irradiation parameters, and efficacy assessment, provides a valuable reference for the design of subcellular PDT. The steps for promoting clinical translation involve strict screening of biocompatible, stable nanocarriers (material level), comprehensive evaluation of tumor targeting/killing (cellular level), in-depth *in vivo* pharmacokinetic and toxicity studies (animal level), and well-designed multi-center trials (clinical level). Collaborating with regulators to develop tailored approval standards will accelerate translation of photosensitizers to clinical use.

In terms of emerging trends, interdisciplinary integration drives PDT development. Deep integration with artificial intelligence enables accurate prediction of patient responses to PDT by building tumor models and analyzing clinical data, optimizing light parameters for personalized treatment based on tumor heterogeneity. Combining with immune checkpoint inhibitors creates synergy: PDT releases tumor-associated antigens to activate immunity, while inhibitors enhance the immune system’s ability to recognize and eliminate tumor cells, strengthening systemic anti-tumor immunity. Technically, activatable photosensitizers remain inert in normal physiology but activate to generate ROS upon sensing tumor microenvironment signals (*e.g.*, low pH, high ATP, specific enzymes), achieving precise tumor killing. Multimodal platforms integrate imaging, PDT, and PTT, with real-time imaging monitoring tumor location, size, and treatment changes to guide therapies and improve efficacy. Additionally, PDT’s applications extend beyond tumors, encompassing effective killing of bacteria, fungi, and viruses (especially drug-resistant strains) without inducing resistance, as well as clearing abnormally aggregated proteins and reducing neuroinflammation in neurodegenerative diseases.

In conclusion, by deeply exploring subcellular targeting mechanisms and combining advanced materials science with biological insights, the performance of PDT is expected to be further optimized. Especially under the premise of overcoming the above challenges, accelerating clinical translation, and keeping up with emerging trends, subcellular organelle-targeted PDT is expected to break through the limitations of existing clinical treatments in the future, achieve breakthrough expansion of clinical indications, gradually expand from the current treatment of some cancers to more disease fields, transition to a wider range of clinical applications, and make greater contributions to the cause of human health.
